# To Carbon or Not to Carbon: Rethinking Electrode Design in Unitized Reversible Fuel Cells

**DOI:** 10.1021/acsami.5c15144

**Published:** 2026-03-03

**Authors:** Mahmoud M. Gomaa, Prince S. A. Nopuo, Manuel Andrés Rodrigo, Justo Lobato

**Affiliations:** † Physics Department, Faculty of Science, 68843 Minia University, P.O. Box 61519 Minia 61519, Egypt; ‡ Chemical Engineering Department, Enrique Costa Novella building, 16733 University of Castilla-La Mancha, Av. Camilo José Cela 12, 13004 Ciudad Real, Spain

**Keywords:** microporous layer, unitized reversible fuel cell, chlor-alkali electrolysis, carbon-based electrodes, system efficiency, energy storage

## Abstract

The development of efficient and scalable energy storage systems remains a major challenge in the transition to renewable energy. Unitized reversible fuel cells (URFCs), capable of operating in both electrolysis and fuel cell modes, offer a promising solution. In this context, integrating the chlor-alkali process into URFCs enables not only cost-effective energy storage but also environmental benefits such as CO_2_ capture via alkaline absorption. While chlor-alkali electrolysis is well established, the reversible operation is not well known. This study addresses a key design question: the role of carbon-based materials in electrode architecture, specifically in the use of a carbon-based microporous layer. Titanium felt electrodes were modified with microporous layers (MPLs) containing 1, 2, and 3 mgC/cm^2^ and coated with a RuO_2_–Pt catalyst using a Pechini-type polymeric precursor method. The results showed that increasing the carbon content, the electrode resistance was reduced and surface hydrophobicity was enhanced, achieving the best results with 2 mgC/cm^2^ in the MPL. Moreover, in electrolysis mode, the hydrogen production efficiency improved with temperature, reaching 15 mgH_2_/Wh at 60 °C (surpassing industrial benchmarks). The system also achieved high Faradaic efficiency for hydrogen production (>98%) and enabled simultaneous CO_2_ capture via cathodic alkaline absorption. In fuel cell mode, the optimized electrode reached a peak power density of ∼30 mW/cm^2^ at 60 °C, an order of magnitude higher than previously reported in the literature for similar systems. The results are very promising and position chlor-alkali-based reversible electrochemical cells as a promising platform for efficient, scalable, and multifunctional energy storage and conversion technologies.

## Graphical abstract

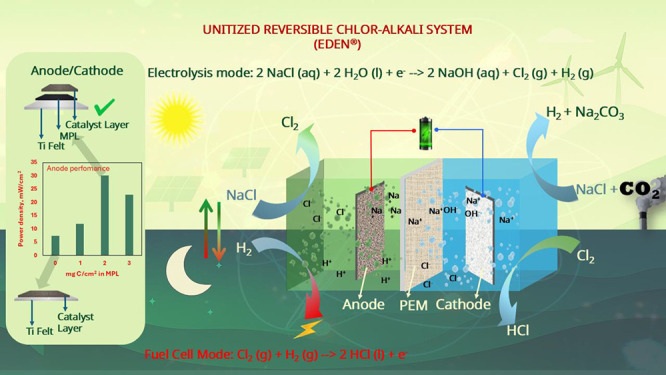

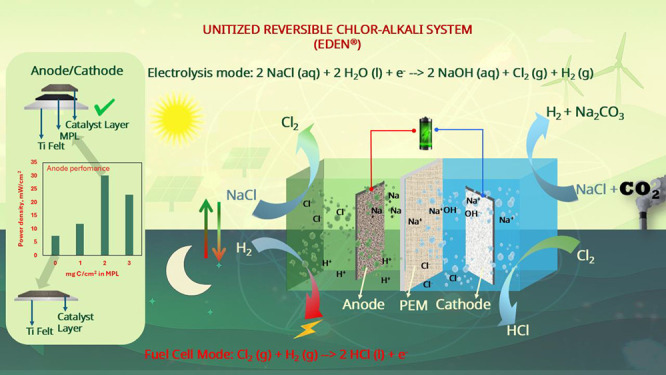

## 1 Introduction

The temperature on the Earth’s surface has increased more during the last 30 years than during the last century. Moreover, the carbon dioxide concentration in the atmosphere has also increased exponentially during the last years, as can be seen in the Keeling curve, which records the atmospheric CO_2_ from the Mauna Loa Observatory.^1^ In this scenario, the reduction of carbon dioxide emissions is mandatory. Thus, the reduction in the use of fossil fuels and the increase in the use of renewable energy sources are very important. Nevertheless, renewable energy sources are not always available when they are needed, and hence, renewable energy storage systems are required. Recently, our research group has developed the EDEN technology, which is a renewable energy storage system based on the chlor-alkali electrolysis, besides the carbon dioxide capture processes.^2,3^ This technology produces chlorine species in the anode, whereas in the cathode, hydrogen and sodium hydroxide are produced. The last one can be used as an absorber to fix the carbon dioxide through the well-known operation unit of the absorption process.^4^

At the beginning, the chlor-alkali industry relied on diaphragm and mercury electrochemical cells. Nowadays, the cation exchange membrane-based electrochemical cells reach more than 80% of global chlor-alkali capacities because of the lower energy consumption.^5^

The aim of this work is not only to study chlor-alkali electrolysis based on a cation exchange membrane but also to assess the possibility of using it as a unitized reversible electrochemical cell for energy storage. Thus, during the peak power, the electrochemical cell would be operating in electrolysis mode, producing chlorine, hydrogen, and sodium hydroxide according to the following eqs 1 to 3.

1 $$a\mathrm{node}:\;2{\mathrm{Cl}}_{\left(\mathrm{aq}\right)}^{-}\to {\mathrm{Cl}}_{2\left(g\right)}+2e^{-},E^{{}^{\circ}}=+1.358\;V$$

2 $$c\mathrm{athode}:2H_{2}O_{\left(l\right)}+2e^{-}↔H_{2\left(g\right)}+2{\mathrm{OH}}_{\left(\mathrm{aq}\right)}^{-},E^{{}^{\circ}}=-0.828\;V$$

3 $$t\mathrm{otal}:\;2H_{2}O+2\mathrm{NaCl}\to 2\mathrm{NaOH}+{\mathrm{Cl}}_{2}+H_{2},E^{{}^{\circ}}=2.19\;V$$

Chlorine and hydrogen should be stored during the day and during the night, or when energy demand is high, the electrochemical cell operates as a fuel cell according to the equations from [Disp-formula eq4] to 6:

4 $$a\mathrm{node}:\;H_{2}\to 2H^{+}+2e^{-},E^{{}^{\circ}}=0.000\;V$$

5 $$c\mathrm{athode}:\;{\mathrm{Cl}}_{2}+2e^{-}↔2{\mathrm{Cl}}_{\left(aq\right)}^{-},E^{{}^{\circ}}=+1.358\;V$$

6 $$t\mathrm{otal}:\;H_{2}+{\mathrm{Cl}}_{2}\to 2\mathrm{HCl},E=1.36\;V$$

The main part of the unitized reversible electrochemical cell is the membrane electrode assembly (MEA), as it is for proton exchange membrane electrolysis cells (PEMECs) or fuel cells (PEMFCs). There are numerous types of electrodes that have been used in chlor-alkali electrolysis. For example, Ti mesh has been used for a membraneless electrolyzer where catalysts based on silver or sodium–manganese oxide are deposited. Moreover, gas diffusion electrodes have been tested based on graphite,^6,7^ nickel foam, or titanium.^3,8^ Zhang et al. have recently published an interesting review where it is pointed out that new electrodes must still be prepared with low price and high durability under the extreme conditions that are reached in the chlor-alkali electrolysis.^9^ From the literature review, it can be noticed that the typical electrodes consist mainly of macrostructure-based materials such as metal foam, mesh, or plate,^10^ where the electrocatalytic layer is deposited on top of these materials. This catalytic layer is composed of nanometer- to micrometer-sized particles and their agglomerates. When the electrode is porous, the fine catalyst particles can penetrate the body of the macroporous electrode, where they are not adequately connected to the membrane, and hence, the three-phase boundary is not promoted. If the catalytic layer is not perfectly continuous, there is a significant risk of creating areas without any contact with the anode or cathode.^11^ These drawbacks have been solved by employing a microporous layer (MPL) for PEMFCs,^12^ and some attempts have been carried out for PEMECs.^13^

The most used materials for the MPL for PEMFCs or PEMECs are carbon-based,^14^ but other materials such as SiC^15^ and IrO_2_ ^11^ have been tested or even Ti powder deposited on a titanium felt electrode for a unitized reversible fuel cell.^16^

Thus, in this work, MPL with different carbon contents deposited on titanium felt electrodes is going to be evaluated for the first time for the renewable energy storage system using a unitized reversible PEMFC based on the chlor-alkali process, i.e, an electrochemical cell that can operate in both electrolysis and fuel cell mode, such as redox flow batteries. Moreover, potential CO_2_ fixation with this system will also be evaluated.

## 2 Experimental Section

### 2.1 Materials

Titanium felt (Bekaert, 0.29 ± 5 mm thickness) was used as received; no platinization or noble metal coating. Ti felt was used as the substrate for the reversible electrode. Hydrochloric acid (HCl, 37%) and oxalic acid (C_2_H_2_O_4_, 99.5%) were obtained from Sigma-Aldrich and used for chemical pretreatment of the titanium substrate. Carbon black (Vulcan XC-72R) and a 10% poly­(tetrafluoroethylene) (PTFE) suspension were used to prepare the microporous layer (MPL).

Ruthenium­(III) chloride hydrate (RuCl_3_·3H_2_O, Alfa Aesar, 38 wt % Ru) and hexachloroplatinic acid hydrate (H_2_PtCl_6_·7H_2_O, Merck, 40 wt % Pt) were used as metal precursors for RuO_2_ and Pt catalyst synthesis, respectively. Ethylene glycol (C_2_H_6_O_2_, ≥99%, Sigma-Aldrich) and citric acid (C_6_H_8_O_7_, ≥99.5%) were obtained from Sigma-Aldrich. Nafion ionomer (5 wt % dispersion in alcohol/water, Sigma-Aldrich) was used as the binder in catalyst inks.

Isopropanol (C_3_H_8_O, 99.8%, Sigma-Aldrich) was used as the solvent in all ink formulations. Additional reagents included sodium chloride (NaCl, Panreac, 99.5%) and hydrochloric acid (HCl, Neon, 38.0%).

For the hydrogen electrode, a commercial carbon cloth (FuelCellStore) was used as the gas diffusion layer. Commercial platinum on carbon catalyst (Pt/C, 40 wt % Pt) was used for the hydrogen side.

### 2.2 Preparation of the Chlorine Electrode

This electrode, designed to function in both the chlorine evolution reaction (CER) during electrolysis and the chlorine reduction reaction (CRR) during fuel cell operation, was fabricated by using titanium felt with a total surface area of about 4 cm^2^ as the substrate. To activate the surface and remove contaminants, the titanium substrate underwent a two-step acid treatment: immersion in 20% (w/w) hydrochloric acid followed by 10% (w/w) oxalic acid, each for 15 min at 80 °C. The treated substrates were then rinsed thoroughly with Milli-Q deionized water and dried at 130 °C.

A microporous layer (MPL) was applied onto the cleaned titanium substrate by air-spraying a dispersion containing Vulcan XC-72R carbon: PTFE solution mass ratio of 90:10 (w/w). MPL loadings of 1, 2, and 3 mg of C/cm^2^ were used to study the effect of carbon content on electrode performance. The coated substrates were subsequently dried and thermally treated at 130 °C for 1 h to enhance mechanical stability and adhesion.

Catalyst deposition was carried out using a polymeric precursor method (Pechini method)^17^ to produce a composite layer containing 75 wt % RuO_2_ and 25 wt % Pt. The precursor solution was prepared by dissolving citric acid in ethylene glycol at 60 °C using a molar ratio of 3:10 (CA: EG) as chelating and polymerizing agents, respectively. Stoichiometric amounts of RuCl_3_·3H_2_O (Aldrich) and H_2_PtCl_6_·7H_2_O (Aldrich) were added to this solution to achieve the desired metal composition. The resulting solution was brushed onto the coated titanium felt with the MPL.

The coated electrodes underwent a multistep thermal treatment to promote oxide formation and eliminate organic components: 130 °C for 10 min (drying), 250 °C for 20 min (decomposition), and 400 °C for 30 min (crystallization). This deposition–calcination cycle was repeated ten times to achieve a final catalyst loading of approximately 0.45 mg/cm^2^. After the final heating step, the electrodes were cooled to room temperature at a controlled rate of 5 °C/min.

### 2.3 Preparation of the H_2_ Electrode

The hydrogen-side electrode with an area of 4 cm^2^ was prepared by using commercial carbon cloth as the substrate. A platinum catalyst supported on carbon (Pt/C, 40 wt % Pt) was dispersed in isopropanol and mixed with a 5 wt % Nafion solution to form a homogeneous catalyst ink. The ink was applied to the carbon cloth via air-spraying to achieve a platinum loading of 0.2 mg/cm^2^. After deposition, the electrode was dried at 80 °C and stored under ambient conditions until use.

### 2.4 Electrochemical Cell Design

A custom-designed electrochemical cell was fabricated by using three-dimensional (3D) printing. The design included two separate chambers: one for the anolyte and the other for the catholyte. A central channel was integrated to hold the membrane electrode assembly (MEA), which was configured in a zero-gap arrangement to ensure direct contact between the anode, membrane, and cathode, thereby minimizing the ohmic resistance. The membrane used in this study was Nafion 117, which was pretreated through a standard activation procedure to enhance its ionic conductivity and performance.^18^ Although Nafion 117 is supplied in the H^+^ form, during electrolysis in NaCl electrolyte, the membrane undergoes in situ Na^+^/H^+^ exchange at the anode side (−SO_3_^–^·H^+^ + Na^+^ ⇌ −SO_3_^–^·Na^+^ + H^+^), enabling the Na^+^ transport required for charge balance and NaOH formation. In fuel-cell operation, the acidic environment reprotonates the membrane, yielding a mixed H^+^/Na^+^ profile that supports reversible operation in both modes.

Both electrodes were engineered to function reversibly, operating as either the anode or cathode depending on the cell’s mode. Specifically, the electrode facilitating chloride oxidation during electrolysis served as the cathode for chlorine reduction during the fuel cell operation. A schematic diagram of the assembled cell is presented in Figure 1.

**Figure 1:**
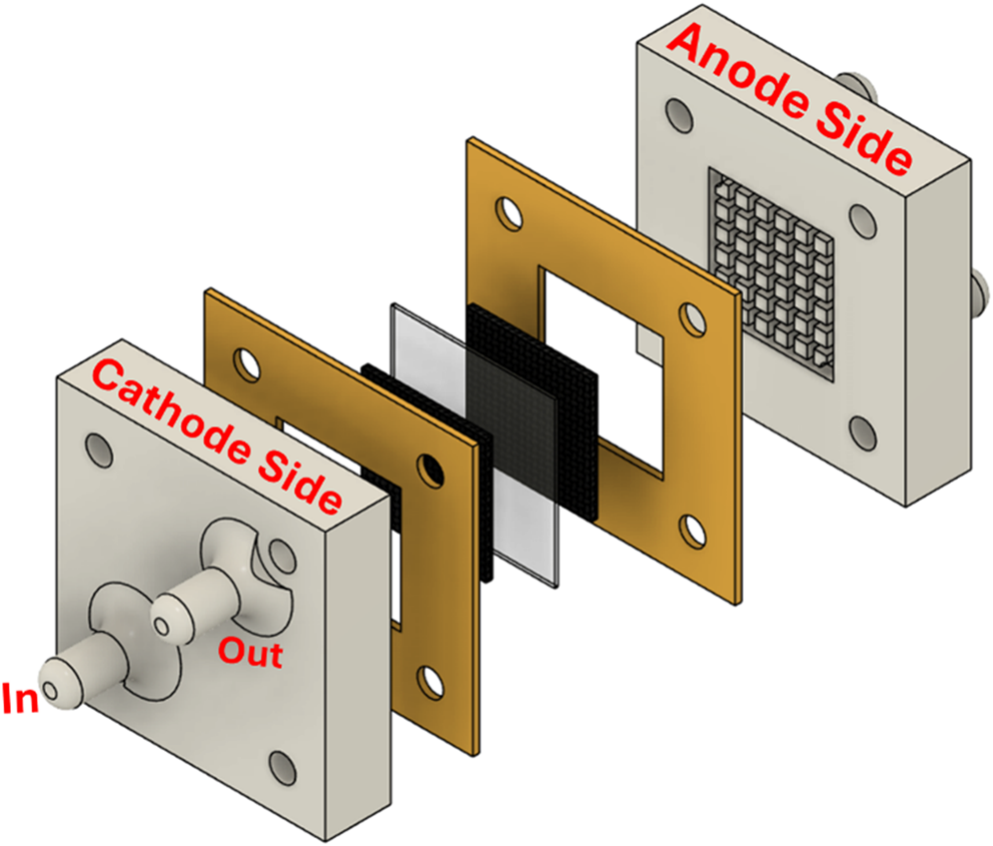
Schematic diagram of the custom 3D-printed dual-compartment electrochemical cell, showing the anolyte and catholyte chambers, the central membrane electrode assembly (MEA), and the zero-gap configuration for reversible operation in both electrolysis and H_2_/Cl_2_ fuel cell modes.

During the electrolysis mode, a 2 M NaCl brine solution was continuously circulated through both the anodic and cathodic compartments of the electrochemical system at a flow rate of 30 mL/min. Oxidation of chloride ions (Cl^–^) occurred at the anode, leading to the generation of Cl_2_, which dissolved into the surrounding NaCl solution to form HClO via hydrolysis, whereas at the cathode, water molecules were reduced to yield H_2_. Simultaneously, sodium ions (Na^+^) migrated toward the cathode and reacted with hydroxide ions (OH^–^), which were formed during the reduction process, resulting in the production of NaOH, as described by eqs 1 and 4. The electrolysis mode was conducted at three different currents (0.2, 0.4, and 0.6 A) across a range of temperatures (20 °C, 40 °C, 60 °C, and 80 °C). At each temperature, the system was operated for a fixed duration of 2 h to evaluate the thermal influence on cell performance and stability.

In the fuel cell operation mode, dry hydrogen gas was supplied into the anode at a controlled rate of 50 mL/min using a compressed gas cylinder. Concurrently, a commercial HClO solution from Bosque Verde, Spain, specifically a standard bleach formulation, was pumped to the cathode at a flow rate of 30 mL/min. The electrochemical system was operated for 1 h at 0.5 V, and then polarization curves and impedance analysis were carried out at different temperatures.

Electrochemical impedance spectroscopy (EIS) was conducted using the established electrochemical cell setup, with measurements spanning frequencies from 50 Hz to 100 kHz. Polarization behavior was characterized via an AUTOLAB/PGSTAT 302N potentiostat/galvanostat system (Metrohm Autolab). Simultaneously, hydrogen generation rates were continuously monitored during the experimental runs. Additionally, pH measurements were performed using a GLP22 pH meter (Crison Instruments) to monitor the acidity levels throughout the experimental procedures.

### 2.5 Contact Angle Measurement

The static contact angle of deionized water droplets on the sample surfaces was measured by using an Attension Theta Flex optical tensiometer (Biolin Scientific) operated with OneAttension software. All measurements were conducted under ambient laboratory conditions.

### 2.6 Electrical/EIS Measurements

#### 2.6.1 Ex Situ Electrical Resistance (Four-Point Probe)

MPL-coated Ti felt specimens without catalysts were characterized with a four-point probe to determine the in-plane electronic resistance of the Ti felt/MPL plate. Resistance values are reported as *R* (Ω) obtained from the linear current/voltage response (Ohm’s law). This measurement isolates the electronic conductivity of the felt/MPL prior to catalyst deposition and membrane assembly, independent of electrolyte/MEA effects present during electrolysis.

#### 2.6.2 In Situ Electrochemical Impedance Spectroscopy (EIS)

EIS was performed on the full electrochemical cell with MEA configuration, including both electrodes (anode, cathode with catalyst layers) and membrane (Nafion 117) under electrolysis operating conditions in a through-plane configuration. In the Nyquist representation (imaginary vs real impedance), the high-frequency intercept on the real axis yields the ohmic resistance, which reflects the whole-cell series resistance, i.e., the sum of membrane/ionomer ionic resistance and interfacial/contact resistances. This measurement quantifies the total through-plane ohmic losses of the operating MEA during electrolysis. The EIS measurements were performed over a frequency range of 50 Hz–100 kHz in the same electrochemical cell setup.

### 2.7 Scanning Electron Microscopy

The morphology and microstructure of the electrodes were analyzed by using a field emission scanning electron microscope (FE-SEM, GeminiSEM 500, Oberkochen, Germany). The device was used to conduct SEM imaging, elemental mapping, and energy-dispersive X-ray spectroscopy (EDS) analyses, operating in high-vacuum mode at an accelerating voltage of 15 kV and without a metal coating. For the elemental composition analysis, the FE-SEM instrument was equipped with an EDS 80 mm2 detector at 30 kV (Oxford Instruments, Abingdon, United Kingdom). The samples were placed and mounted on standard aluminum SEM holders with carbon adhesive tabs.

## 3 Results and Discussion

### 3.1 Ohmic Resistance of Electrode Materials

To study the intrinsic electrical resistance of the electrode structures, four-point probe measurements were conducted on Ti felt electrodes with different MPL loadings (0, 1, 2, and 3 mg of C/cm^2^) without the catalyst layer, as shown in Figure 2a. The linear *I*–*V* relationships confirm purely ohmic behavior across all electrodes.

**Figure 2:**
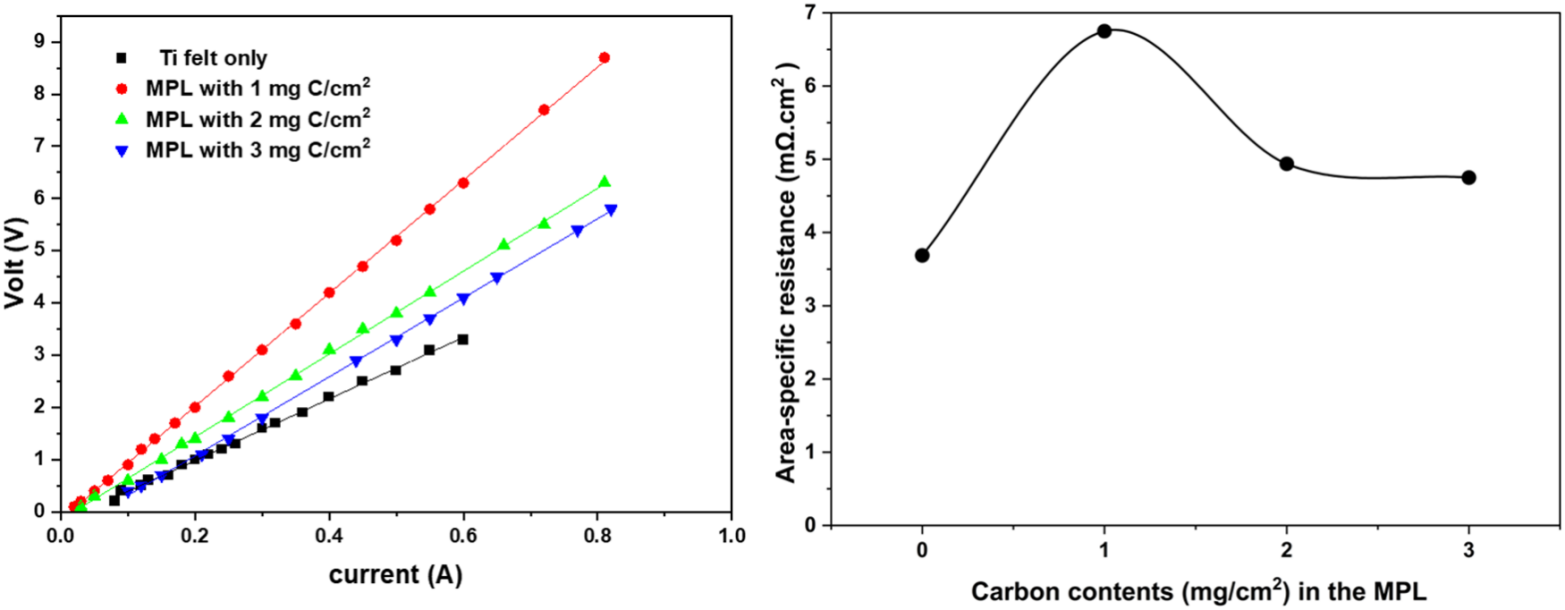
Linear *I*–*V* plots and the corresponding area-specific resistance values from four-point probe measurements of Ti felt electrodes varying carbon content in the MPL (1 mg C/cm^2^, 2 mg C/cm^2^, 3 mg C/cm^2^).

From the *I*–*V* curves, the resistance (Figure 2b) was calculated based on the slope (*R* = Δ*V*/Δ*I*) in Figure 2a. The Ti felt without MPL showed an area-specific resistance of approximately 3.6 mΩ cm^2^, while the area-specific resistance increased to 6.75 mΩ cm^2^ with 1 mg of C/cm^2^ in the 3MPL. Interestingly, resistance then decreased with further loading of carbon in the MPL: 4.9 mΩ cm^2^ for 2 mg of C/cm^2^ and 4.7 mΩ cm^2^ for 3 mg of C/cm^2^ in the MPL.

This nonlinear trend indicates that a small amount of carbon in the MPL (1 mg) may increase interfacial resistance due to incomplete surface coverage or poor contact between the MPL and the Ti substrate, possibly introducing microstructural gaps that hinder current flow. Such effects have been noted in systems where initial catalyst layer deposition creates heterogeneity or surface disruption.^19^

However, higher carbon loadings (2–3 mg/cm^2^) result in more uniform and continuous coverage, enhancing the interface between the MPL and the Ti substrate and hence improving the electrical connectivity and enabling better current distribution through the electrode. The observed 4.7 mΩ cm^2^ resistance at 3 mg C/cm^2^ in the MPL, the lowest of all tested configurations, reflects the improved conduction path provided by the densified and integrated MPL.

These results support the conclusion that although thin MPL layers may initially hinder electrical performance, optimized MPL thickness improves not only mass and gas transport but also bulk electronic conductivity—contributing to more efficient and stable electrochemical operation.^20^

### 3.2 Contact Angle Measurements

Figure 3 illustrates the contact angle measurements performed on the electrodes with different carbon loadings of MPL. The contact angles for all MPL-containing electrodes were >140°, confirming their hydrophobic nature. Notably, the electrode with 2 mgC/cm^2^ in the MPL exhibited the highest contact angle, approximately 152.3°, indicating a high hydrophobicity degree. This suggests that at this loading, the surface microstructure achieves an optimal balance between porosity and roughness, enhancing water repellency, which is a crucial characteristic for maintaining gas transport and minimizing electrolyte flooding in electrochemical systems.^21^

**Figure 3:**
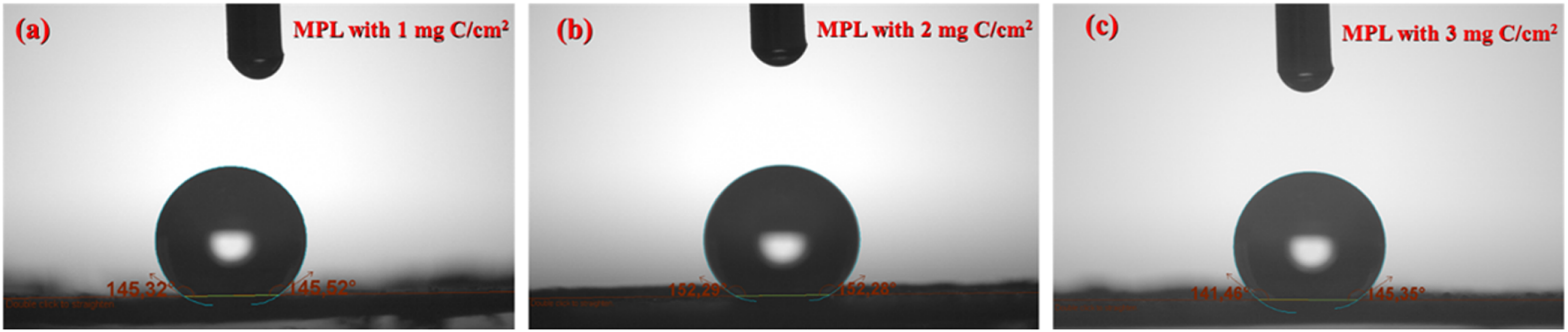
Contact angle images of water droplets on electrodes coated with different carbon loadings in the MPL: (a) 1 mg of C/cm^2^, (b) 2 mg of C/cm^2^, and (c) 3 mg of C/cm^2^.

In the case of the electrode with 1 mg C/cm^2^ in the MPL, the contact angle was slightly lower (∼145°), suggesting partial surface exposure or insufficient coverage. Moreover, at 3 mg C/cm^2^ based MPL, the angle dropped modestly to the 143–145° range, likely due to excess MPL deposition, which can fill pores or alter surface texture, thereby compromising the hierarchical roughness that enhances hydrophobicity.^22^

It is important to highlight that the contact angle measurements could not be performed on the electrode without MPL, as the porous titanium felt substrate allowed water droplets to be absorbed or dispersed into the internal structure rather than to form a measurable droplet on the surface. This observation further underscores the functional role of the MPL in imparting controlled surface properties and maintaining the interfacial separation between the liquid electrolyte and gaseous products.

### 3.3 Scanning Electron Microscopy

The surface morphology of the prepared electrodes was systematically examined by SEM and is presented in Figure 4. Clear morphological changes are observed in the Ti electrodes as the carbon content in the microporous layer (MPL) increases. The Ti felt electrode without MPL (Figure 4a) shows an open fibrous network with large, interconnected pores, indicating a highly porous and uncoated surface. Upon the introduction of 1 mg of C/cm^2^ MPL (Figure 4c), the fibers become partially covered with fine particles, increasing surface roughness while maintaining visible pore channels. At 2 mg of C/cm^2^ (Figure 4e), a denser and more uniform coating forms, with most fibers partially embedded in the deposited layer, reducing pore size. The 3 mg of C/cm^2^ electrode (Figure 4g) exhibits a thick and continuous granular coating that nearly obscures the underlying structure, leading to a pore blockage and reduced open spaces. Overall, increasing MPL loading raises surface roughness and particle coverage. The thickness measurements from the cross sections support this progression. Using the Ti felt only (≈290 μm) as the reference, total section thicknesses are ≈293.6 μm (1 mg C/cm^2^), 303 μm (2 mg C/cm^2^), and 423 μm (3 mg C/cm^2^), giving apparent overlayer additions of ∼6 μm, ∼12 μm, and ∼133 μm, respectively.

**Figure 4:**
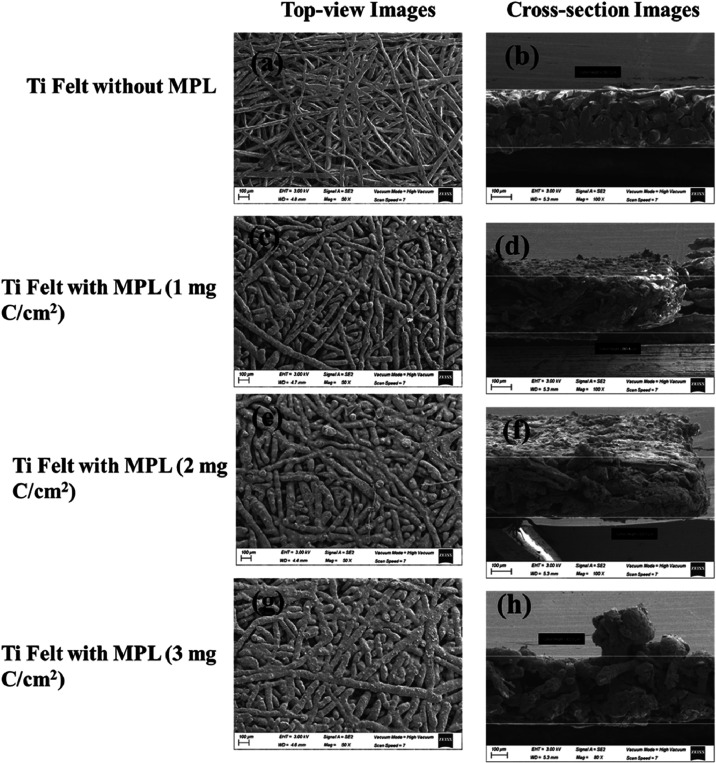
SEM of Ti felt electrodes: (a) top view of no MPL, (b) cross-section of no MPL, (c) top view of 1 mg C/cm^2^ MPL, (d) cross-section of 1 mg C/cm^2^ MPL, (e) top view of 2 mg C/cm^2^ MPL, (f) cross-section of 2 mg C/cm^2^ MPL, (g) top view of 3 mg C/cm^2^ MPL, and (h) cross-section of 3 mg C/cm^2^ MPL. All SEM images were acquired at an accelerating voltage of 3.0 kV using a secondary electron (SE2) detector at a scan speed of 7. Top-view images were recorded at 50× magnification, and cross-sectional images were recorded at 100× magnification except for the sample with a 3 mg C/cm^2^ MPL. In all cases, the bar scale corresponds to 100 μm.

The microporous layer (MPL) employed in this study is defined based on its functional role within the electrode architecture rather than strict morphological uniformity. In electrochemical energy systems, an MPL refers to a carbon/PTFE-based porous interlayer positioned between a macroporous transport substrate and the catalyst layer, serving to improve electrical contact, control wettability, facilitate gas transport, and promote a uniform current distribution. While the MPL morphology observed here is less uniform than that typically reported for conventional PEM fuel cells, this difference arises from the use of a titanium felt substrate and from the specific requirements of chlor-alkali and unitized reversible operation. Importantly, the layer fulfills the defining functional characteristics of an MPL, and the term is therefore retained throughout the manuscript for consistency with established usage in the literature.

### 3.4 Polarization Curves at Different Temperatures and with Anodes of Varying Carbon Content in the MPL

Figure 5 presents the behavior of the electrochemical cell in electrolysis mode at various temperatures (20–80 °C) and with anodes having different carbon loadings in the MPL. For all the tests, independently of the carbon content in the MPL, increasing the temperature from 20 to 80 °C consistently results in higher current densities for a given potential. Increasing the temperature decreases both the ohmic resistance and activation overpotentials, resulting in improved electrochemical performance, particularly evident at higher current densities. Moreover, the gas bubble detachment at the electrode surface is more efficient at higher temperatures, which could also contribute to an enhancement in the performance at electrode surfaces.^23^

**Figure 5:**
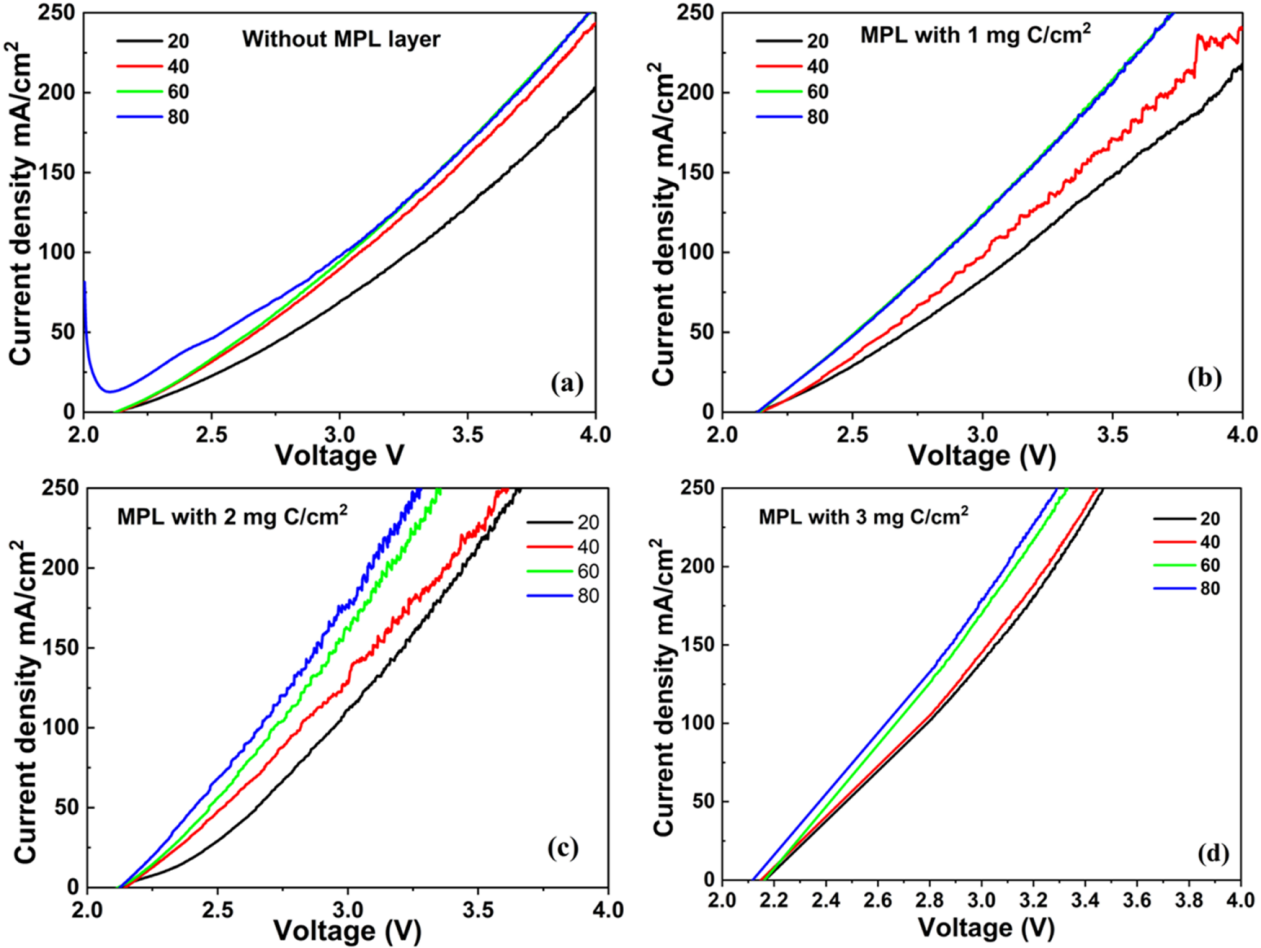
Polarization curves in electrolysis mode at 20 °C, 40 °C, 60 °C, and 80 °C using anodes with different C content in the MPL: (a) no MPL, (b) 1 mg C/cm^2^, (c) 2 mg C/cm^2^, and (d) 3 mg C/cm^2^.

On the other hand, the content of carbon in the MPL of the anode electrodes significantly influences the electrochemical performance at each temperature. It can be noticed that the use of MPL has a positive effect. The higher the carbon content, the better the performance, as the curves generally shift toward higher current densities at the same voltage, indicating improved mass transport and more efficient catalyst utilization. The MPL facilitates better distribution of reactants and enhances the accessibility of electrochemically active sites.^24^ Additionally, it promotes effective gas bubble removal from the electrode surface,^25^ which is critical for maintaining continuous and efficient electrochemical reactions.

### 3.5 Electrochemical Impedance Spectroscopy (EIS)

The EIS results (Figure 6) support the trends observed in the polarization data. For all of the carbon loadings in the MPL, Nyquist plots revealed a consistent decrease in the resistance with increasing temperature. For the electrode without an MPL, impedance remained relatively high across the temperature range, indicating poor electrolyte penetration and inefficient gas management. In contrast, MPL-containing electrochemical reactors exhibited a lower value of resistance. Specifically, the electrodes with 3 mgC/cm^2^ MPL showed the lowest charge transfer resistance and ohmic resistance, confirming enhanced ion mobility and improved interfacial charge transfer kinetics under these conditions.

**Figure 6:**
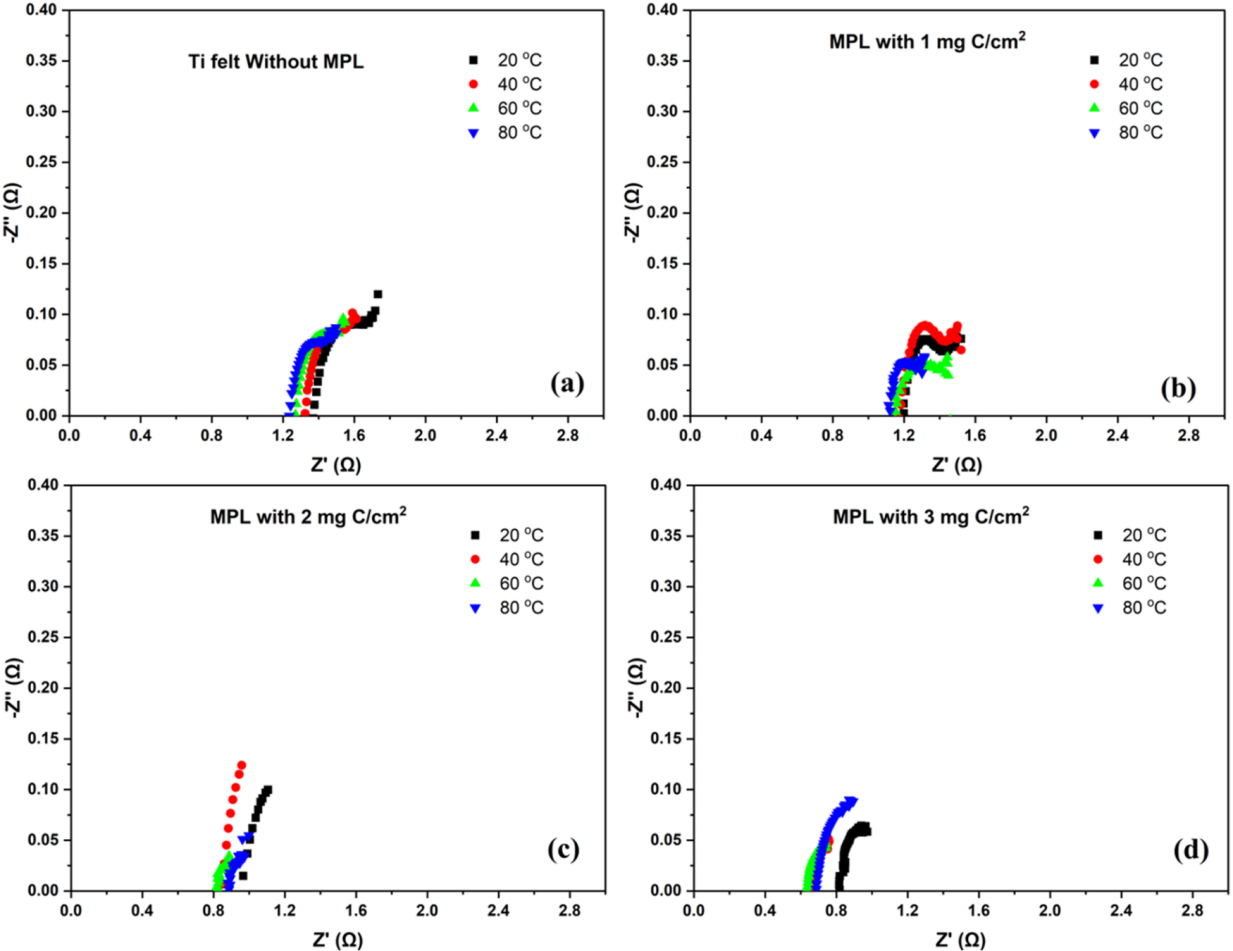
Nyquist plots of Ti felt electrodes with varying carbon loadings in the MPL: (a) no MPL, (b) 1 mg C/cm^2^, (c) 2 mg C/cm^2^, and (d) 3 mg C/cm^2^ at different temperatures (20 °C, 40 °C, 60 °C, and 80 °C) in electrolysis mode.

These impedance characteristics directly explain the polarization behavior: systems with lower impedances showed reduced voltage losses and more stable performance at operation. The reduction in resistance components with temperature also supports the observed decrease in voltage under constant current conditions, affirming that thermal activation significantly enhances the electrochemical kinetics and electrode–electrolyte interaction.

The absolute high-frequency resistance (HFR) values measured in the present system are higher than those reported for state-of-the-art zero-gap water electrolyzers.^26^ This quantitative gap primarily reflects differences in membrane selection, cell architecture, and operational objectives. In this work, Nafion 117 was deliberately selected to enable a unitized reversible system, allowing the same membrane–electrode assembly to operate in both fuel-cell and electrolysis modes. Nafion 117 is substantially thicker and primarily optimized for proton conduction, which is essential for fuel-cell operation, while in electrolysis mode under chlor-alkali conditions, ionic transport involves a significant contribution from Na^+^ ions with intrinsically lower mobility. Consequently, a higher intrinsic ionic resistance is expected compared to specialized, thinner PFSA membranes (e.g., Nafion 900-series) commonly employed in optimized industrial chlor-alkali electrolyzers, where area-specific resistances below ∼0.3–0.5 Ω·cm^2^ are achieved using highly compressed Ti-based porous electrodes.^27^ In addition, although the present cell operates in a zero-gap configuration, the laboratory-scale, 3D-printed design relies on mechanical compression that does not reach the high and uniform hydraulic compression applied in industrial Ti-foam-based cells, leading to increased interfacial contact resistance. Finally, as a unitized reversible fuel cell (URFC), the system must balance the requirements of both electrolysis and fuel-cell operation, inherently introducing trade-offs compared with single-mode industrial electrolyzers optimized solely for minimizing ohmic losses. Nevertheless, at the moderate current densities employed here, the higher absolute HFR does not obscure comparative performance trends but instead provides meaningful insight into the influence of MPL content and electrode architecture on resistance contributions in reversible chlor-alkali systems.

### 3.6 Electrolysis Performance

The response of the electrochemical cell in electrolysis mode under constant current operation was investigated at different temperatures (20 to 80 °C), different carbon contents in the MPL at the anodic side (0, 1, 2, and 3 mg C/cm^2^), and different applied currents (0.2, 0.4, and 0.6 A) is illustrated in Figure 7. The anodic electrode without an MPL exhibited the highest and least stable voltages across all tested conditions. In contrast, using the electrodes modified with an MPL exhibited improved voltage performance and enhanced stability under the same operating conditions. This improvement can be attributed to the structural benefits gained by the MPL. The presence of the MPL improved electrolyte distribution, more efficient ion pathways, better gas management, and provided additional porosity and hydrophobicity, facilitating more effective gas detachment from the electrode surface and promoting uniform ion distribution.^28^ From the EIS Nyquist plots (Figure 6), the high-frequency resistance intercept (*R*) decreases with increasing MPL loading and with increasing temperature, indicating improved interfacial contact and current distribution without blocking ionic access from bulk NaCl.

**Figure 7:**
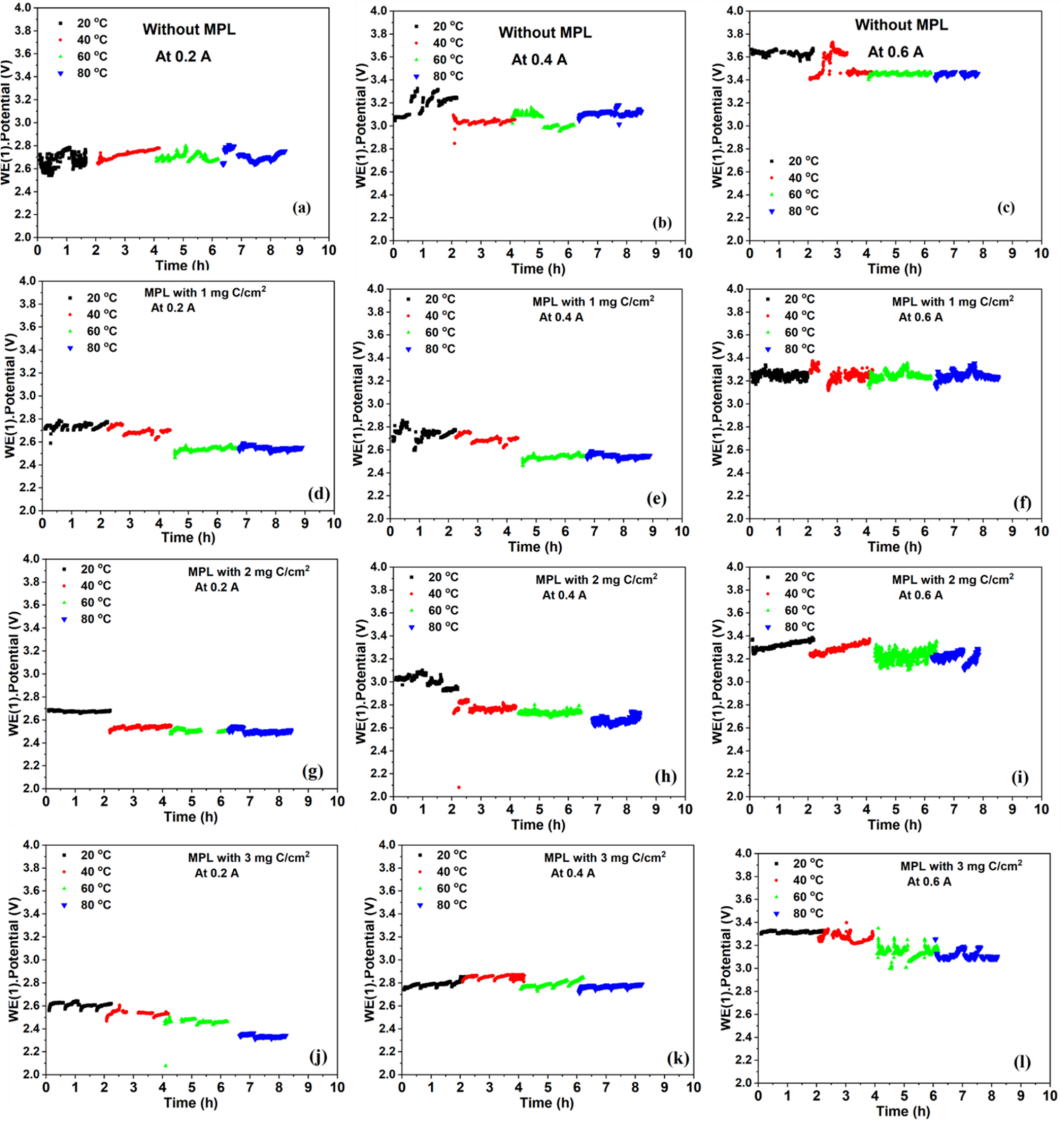
Long-term electrolysis performance of the electrolysis cell at different temperatures (20 °C, 40 °C, 60 °C, and 80 °C) using anodic electrodes with different carbon content in the MPL and different currents in galvanostatic mode: (a) no MPL at 0.2A, (b) no MPL at 0.4A, (c) no MPL at 0.6A, (d) 1 mg C/cm^2^ at 0.2A, (e) 1 mg C/cm^2^ at 0.4A, (f) 1 mg C/cm^2^ at 0.6A, (g) 2 mg C/cm^2^ at 0.2A, (h) 2 mg C/cm^2^ at 0.4A, (i) 2 mg C/cm^2^ at 0.6A, (j) 3 mg C/cm^2^ at 0.2A, (k) 3 mg C/cm^2^ at 0.4A, and (l) 3 mg C/cm^2^ at 0.6A.

Increasing the applied current from 0.2 to 0.6 A resulted in an increase in the voltage values, as was expected (Supporting Figure S1). At higher current values, such as 0.6 A, the rate of electrochemical reactions increases, which in turn increases the rate of chlorine and hydrogen production at the electrode surfaces.^29^ The result was both an increased average voltage and, in some configurations, visible voltage oscillations, particularly under low carbon loading and lower temperature conditions. The high amount of gases at the surface of the electrodes increases the gas bubble accumulation at the electrode–electrolyte interface, which forms a dynamic barrier that reduces the ion access to active sites, thereby increasing the local resistance and causing a reduction in the reaction efficiency.^30,31^

Additionally, in most cases, the operating voltage decreases moderately as the temperature increases, and this trend correlates with our EIS results (Figure 6). Between 20 and 80 °C, the Nyquist plots exhibit a slight leftward shift of the high-frequency intercept (lower Ohmic resistance *R*Ω) and a contraction of the midfrequency semicircle (lower *R*_ct_), indicating improved ionic transport and faster interfacial charge transfer at elevated temperature. The extracted *R*Ω and *R*_ct_ values are compiled in Supporting Table S1 and mirror the polarization curves. Occasional deviations from the expected temperature-dependent trend were observed, especially at elevated temperatures.

The high temperature contributes to the detachment of gas bubbles faster from the electrode surface and minimizes the blockage.^25,32^ However, in some cases, the expected voltage decrease was not clearly observed. This deviation could be attributed to several factors. One of them is that nonuniform wetting of the electrode or membrane at higher temperatures may lead to uneven current distribution and localized resistive hotspots, offsetting the anticipated performance gains.^33^ Moreover, thermal expansion of the electrode materials or MPL may slightly change the porosity or interfacial contact,^34^ which leads to an increase in the resistance in some regions. Additionally, incomplete gas removal or bubble coalescence at the higher rates of gas evolution can reintroduce mass transport limitations.^35^ These results highlight the importance of balancing the current density with structural electrode properties and operational temperature. While higher current densities are desirable for maximizing power output, they must be supported by an optimized electrode architecture and thermal conditions to maintain stable and efficient operation.

Additionally, the time-resolved average voltage behavior for each condition is presented in Figure 8, highlighting how electrode structure and temperature influence operational stability and performance in electrolysis mode. It can be highlighted that the incorporation of a carbonaceous microporous layer in the anode electrode improves the performance of the electrolysis cell. Moreover, a carbon content of 2 mg C/cm^2^ is enough, as the performance of the electrode with 3 mg C/cm^2^ of carbon shows a performance very similar to that of the one with 2 mg C/cm^2^ of carbon.

**Figure 8:**
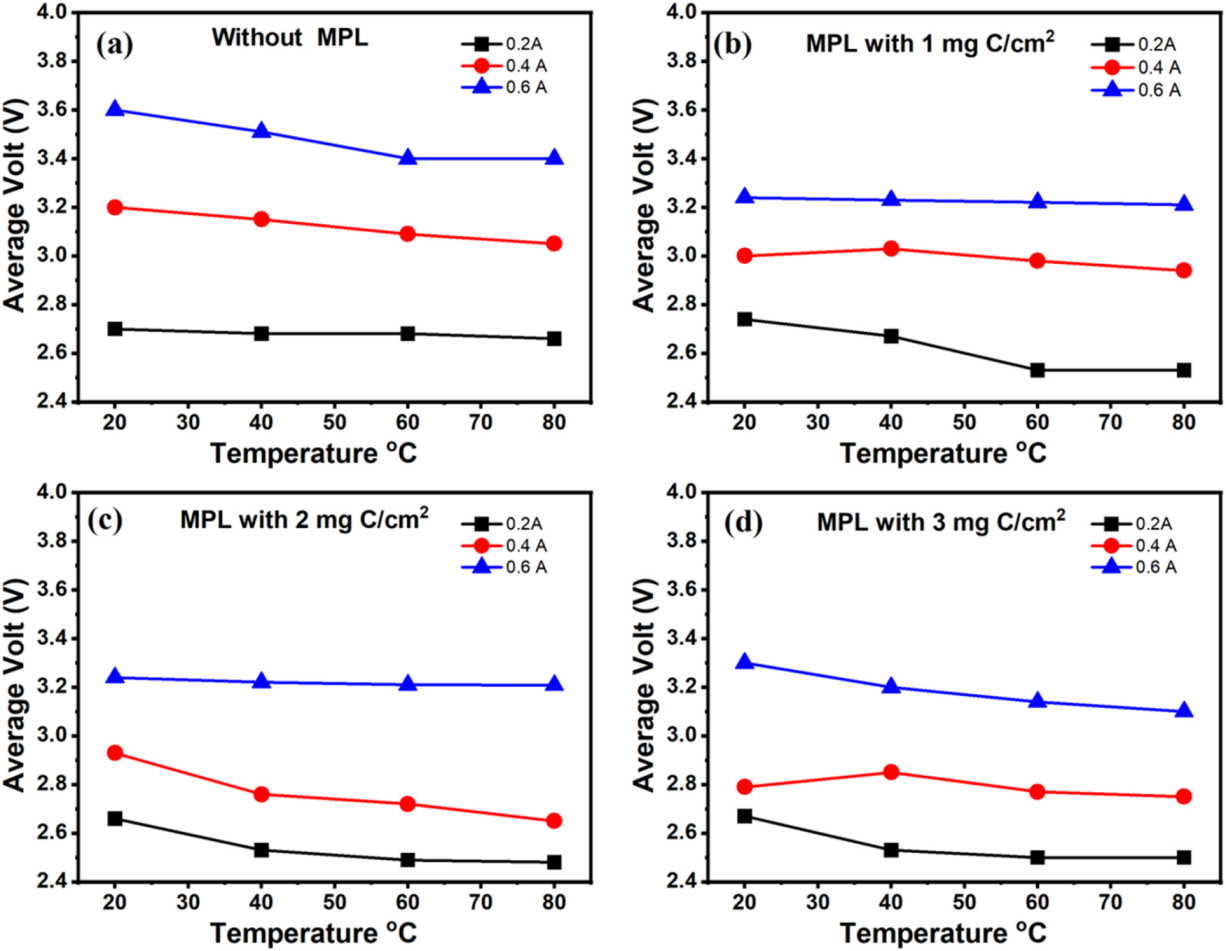
Average cell voltage value as a function of temperature under constant current operation (0.2, 0.4, and 0.6 A) using anodic electrode with different carbon loading in MPL: (a) no MPL, (b) 1 mg C/cm^2^, (c) 2 mg C/cm^2^, and (d) 3 mg C/cm^2^.

Since this electrode operates in a chlorine-evolving environment, the design follows the established dimensionally stable anode (DSA) principle: a RuO_2_–Pt (RuO_2_-rich) catalyst layer on Ti is placed at the reaction front, while the carbon-based MPL is positioned behind the catalyst, where it primarily improves electrical contact, porosity, and wetting. This layer order concentrates the interfacial potential drop and current at the RuO_2_/Ti interface; therefore, the carbon network does not experience the high anodic potentials or reactive intermediates characteristic of the chlorine evolution reaction (CER). This is consistent with the CER literature, where RuO_2_/Ti mixed-oxide anodes are the state of the art for Cl_2_ production due to their low CER overpotential and durability in chloride media, and where carbon is avoided at exposed anode surfaces.^36^ In parallel, PTFE in the MPL raises hydrophobicity and lowers liquid water residence, restricting access of oxidants to carbon sites and supporting effective gas removal behaviors widely reported for PTFE-modified GDL/MPL structures.^37^ Taken together, the material selection and layer order used here (RuO_2_–Pt/Ti at the interface; carbon/MPL with PTFE behind it) offer a robust strategy for anodic operation while retaining the transport and contact benefits of an MPL.

The electrode architecture investigated in this work follows the dimensionally stable anode (DSA) design principle, in which the RuO_2_–Pt catalyst layer is positioned directly at the reaction interface while the carbon-containing layer is located behind the catalyst, away from the primary anodic potential drop. As a result, the highest anodic potentials and the majority of reactive chlorine species generated during the chlorine evolution reaction (CER) are confined to the RuO_2_-rich surface, significantly limiting direct exposure of the carbon phase to oxidizing intermediates. In addition, the incorporation of PTFE in the carbon layer enhances hydrophobicity and restricts electrolyte penetration, further reducing the accessibility of oxidizing chlorine species to carbon sites. Nevertheless, given the highly oxidizing nature of the CER environment, gradual carbon degradation over extended operation time scales cannot be completely excluded. Therefore, while the present results demonstrate stable performance over intermediate durations, extended durability testing and direct probing of the potential distribution within the carbon-containing layer will be required to fully assess the long-term stability.

### 3.7 pH Behavior in Electrolysis Mode

The change in pH at both the cathode and anode during electrolysis provides critical insight into the electrochemical reactions and the role of electrode architecture. Figure 9 shows the pH profiles during electrolysis mode at room temperature under a constant applied current of 0.2 A. As shown in Figure 9, the pH at the cathode increased rapidly from ∼5.5 to above 12.5 within the first 30 min, while the anode pH dropped sharply to values below 2.0. This behavior reflects the characteristic water-splitting reactions: hydrogen evolution at the cathode, which consumes protons and generates hydroxide ions (raising pH), and chlorine evolution at the anode, which generates protons and lowers pH through HClO formation.

**Figure 9:**
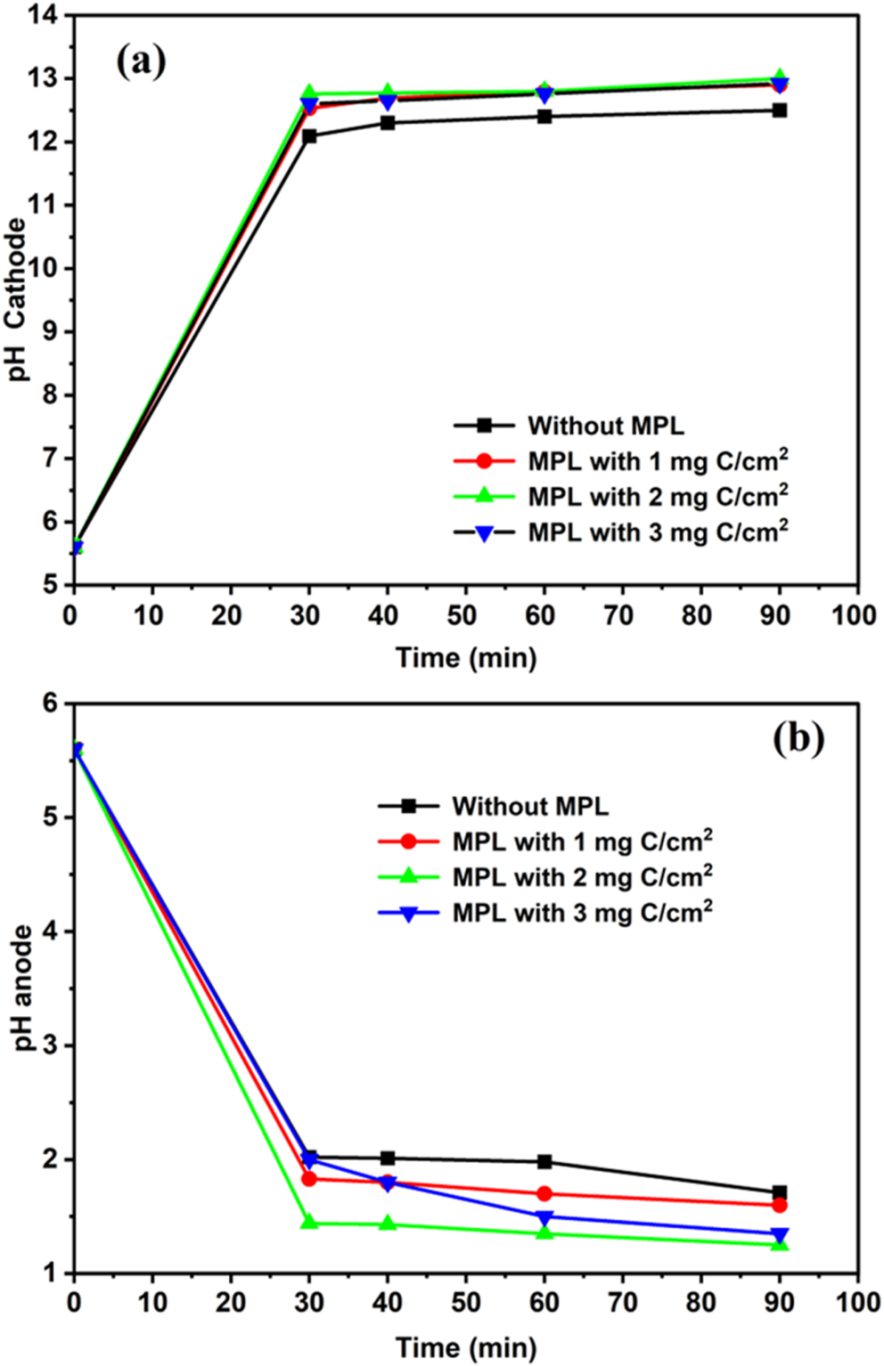
Time-resolved pH profiles during the electrolysis mode at room temperature under a constant applied current of 0.2 A. (a) pH change at the anode compartment. (b) pH change in the cathode compartment.

The presence of the microporous layer (MPL) had a clear impact on the rate and extent of these pH shifts. Electrodes with high carbon content in the MPL (particularly 2 and 3 mg of C/cm^2^) demonstrated more pronounced changes in pH, indicating enhanced electrochemical activity and faster reaction kinetics. This aligns with earlier results where MPL-containing electrodes showed improved voltage stability and lower resistance in both polarization and EIS measurements. The MPL contributes by optimizing gas diffusion and electrolyte distribution, which enhances ionic transport and promotes efficient product removal. This leads to improved reaction kinetics at both the anode and cathode interfaces.

Moreover, the faster drop in pH at the anode side for MPL-modified electrodes suggests improved chlorine evolution efficiency and possibly enhanced wettability and interfacial reactivity. These effects support the next results, where higher MPL loading also resulted in improved hydrogen production efficiency (mg/Wh), indicating that MPL facilitates better utilization of charge and electrolyte composition.

### 3.8 Hydrogen Production

When renewable energy is used, green hydrogen (H_2_) can be produced on the cathodic side during chlor-alkali electrolysis. Figure 10 shows the hydrogen energy efficiency (mgH_2_/Wh) at different conditions (temperature and current densities) for the electrolysis cell equipped with the anode without and with different carbon contents in the MPL. Moreover, the values obtained in this work are compared with those from the literature, as shown in Table 1.

**Figure 10:**
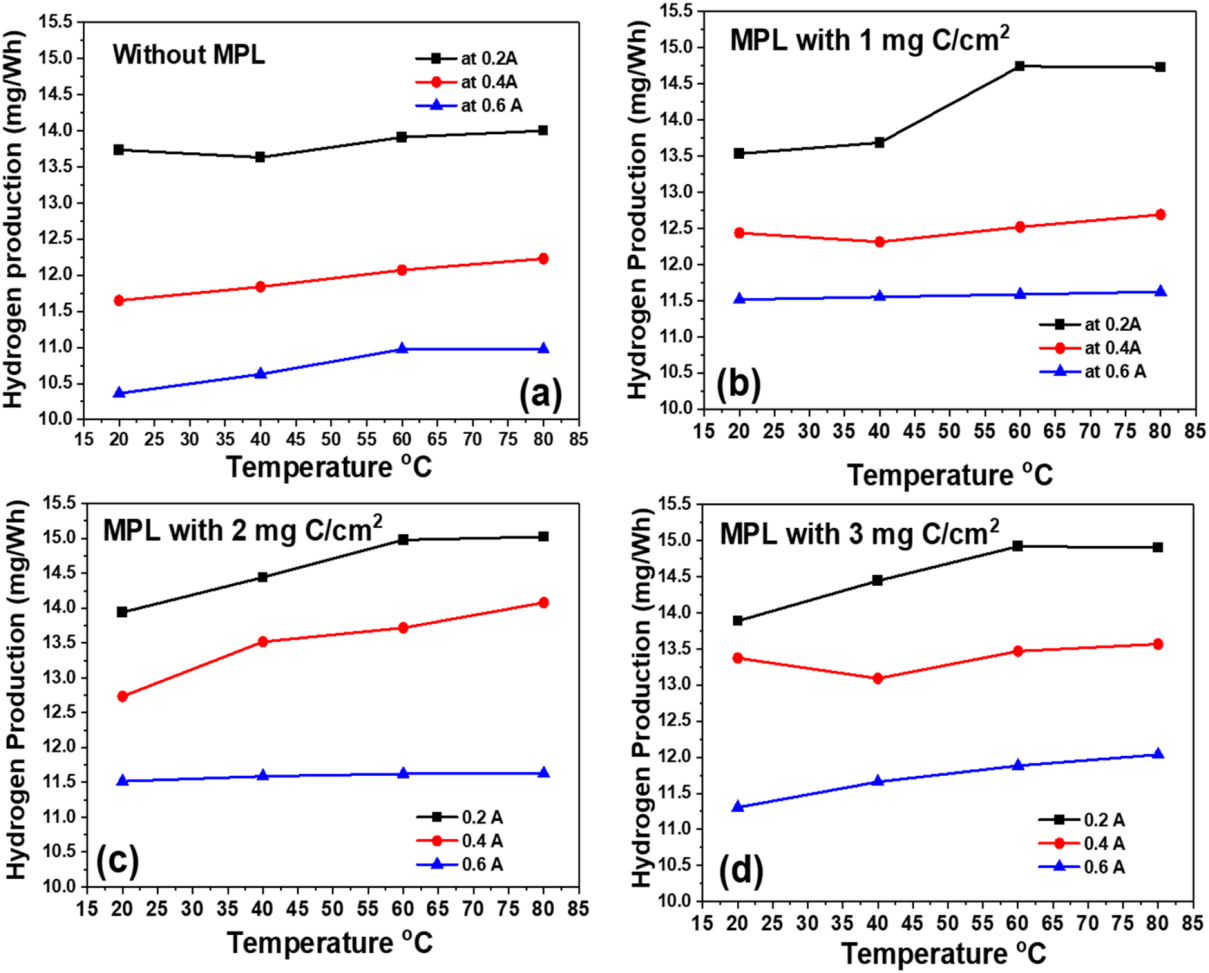
Hydrogen energy efficiency (mgH_2_/Wh) at different current densities using anodic electrodes with MPL with a carbon loading of (a) no MPL, (b) 1 mg C/cm^2^, (c) 2 mg C/cm^2^, and (d) 3 mg C/cm^2^.

**Table 1:** H_2_ Production (mg/Wh) Using Electrolysis Based on Chlor-Alkali Cell Systems

system anode|cathode|membrane	H_2_ production (mg/Wh)	temperature (°C)	current density(mA/cm^2^)	refs
RuO_2_/Pt + MPL with 2 mg C/cm^2^ in Ti felt|Pt/C|nafion	15	60	50	this work
RuO_2_/Pt + MPL with 2 mg C/cm^2^ in Ti felt|Pt/C|nafion	11.5	60	150	this work
3D Ti printed GDE|Pt/C|PFSA	6.1	25	50	Mahmoudian et al.,^3^
graphite or DSA|Pt/C|PFSA membrane	10.63	80–90	400–600	BREF, EU^40^
DSA|Pt/C|nafion	10.6	25	214	Carvela et al.,^41^
RuO_2_/Pt|Pt/C|PVA/Chitosan/PVA membranes	10.8	20	50	Gomaa et al.,^42,43^
RuO_2_/Pt|Pt/C|nafion membrane	9.5	25	50	Romero et al.,^44^
RuO_2_/Pt|Pt/C|Na^+^ form membrane	11	80	50	Gomma et al,^39^

This system achieved Faradaic efficiencies exceeding 98% for hydrogen production (see Supporting, Figures S2 and S3), indicating nearly complete utilization of charge for the desired electrochemical reaction. As illustrated in Figure 10 and Supporting Figure S4, energy efficiency improved with temperature and showed a clear dependence on the content of carbon in the MPL of the anode. The configuration with 2 mg of C/cm^2^ in the MPL consistently showed the highest energy efficiency, indicating optimal electrode microstructure for balancing electronic conductivity, gas diffusion, and electrolyte transport. At low current densities (e.g., 0.2 A), hydrogen production was particularly efficient (∼15 mg/Wh). This is attributed to moderate gas evolution, which allows for unobstructed bubble detachment and preserves the triple-phase boundary, minimizing mass transport resistance and voltage loss. It should be noted that hydrogen production efficiency (mg H_2_/Wh) exhibits an inverse dependence on the current density. Therefore, comparisons across different current densities are not strictly like-for-like and should be interpreted as indicative trends rather than absolute performance metrics.

However, at higher current densities (e.g., 0.6 A), increased hydrogen evolution leads to bubble coalescence and accumulation at the electrode–electrolyte interface. This induces partial blockage of active sites, elevates local ohmic and concentration overpotentials, and reduces the energy efficiency to ∼11.5 mg/Wh. These results are consistent with reported behavior in gas-evolving electrochemical systems, where excessive gas generation impedes charge and mass transfer dynamics.^30^

Comparing the values shown in Table 1, the benchmarked against industrial references such as the EU’s Best Available Techniques Reference Document (BREF) cites a hydrogen production efficiency of about 10.63 mgH_2_/Wh for advanced chlor-alkali systems^6,38^ which is lower than our value obtained under our optimized cell. This highlights that our study exhibits a highly competitive performance.

Importantly, when compared to our previous studies using similar electrodes and membranes, where the hydrogen yield at room temperature was limited to approximately 6.1 mg of H_2_/Wh, the current system demonstrates significant progress. In a recent publication by our group,^39^ a yield of 11 mg H_2_/Wh was achieved, but only at a current density of 50 mA/cm^2^. In contrast, the present work achieves equal or greater efficiency at a much higher current density of 150 mA/cm^2^, indicating substantial improvement in hydrogen production. This advancement is attributed to the enhanced electrode interface structure incorporating a microporous layer (MPL) and the use of elevated operating temperatures, both of which contributed to reduced polarization losses and more effective management of reaction kinetics and product transport.

### 3.9 Fuel-Cell Mode

As outlined in the Introduction, the EDEN technology can be regarded as an energy storage system, where power is initially stored in the form of hydrogen (power-to-chemical) and later utilized as a fuel in a fuel cell to generate electricity. Moreover, when the same device is employed for both charging and discharging, it functions as a redox flow battery or a unitized reversible fuel cell.^39^

When operated in fuel cell mode, the electrochemical cell acted as a H_2_/Cl_2_-based fuel cell. In this configuration, hydrogen gas was supplied from a compressed tank, while chlorine was introduced in the form of hypochlorous acid (HOCl). These reactants facilitated the conversion of stored chemical energy back into electrical energy, enabling the system to function as a fully reversible energy storage unit.

Figure 11 shows the polarization and power density curves of the H_2_/Cl_2_ fuel cell system across a range of temperatures (20 to 80 °C) and for the fuel cell with anodes without and with different carbon loadings in the MPL. Unlike conventional H_2_/O_2_ fuel cells, the H_2_/Cl_2_ system demonstrates distinct electrochemical advantages. Notably, the open-circuit voltage (OCV) for all tested configurations closely aligns with the theoretical value of approximately 1.34 V. This value is based on the standard electrode potentials of the H_2_/H^+^ (0.00 V) and HOCl/Cl^–^ (+1.34 V) redox couples. The close match between experimental and theoretical OCV indicates effective charge separation and minimal internal parasitic losses under open-circuit conditions, underscoring the system’s electrochemical efficiency.

**Figure 11:**
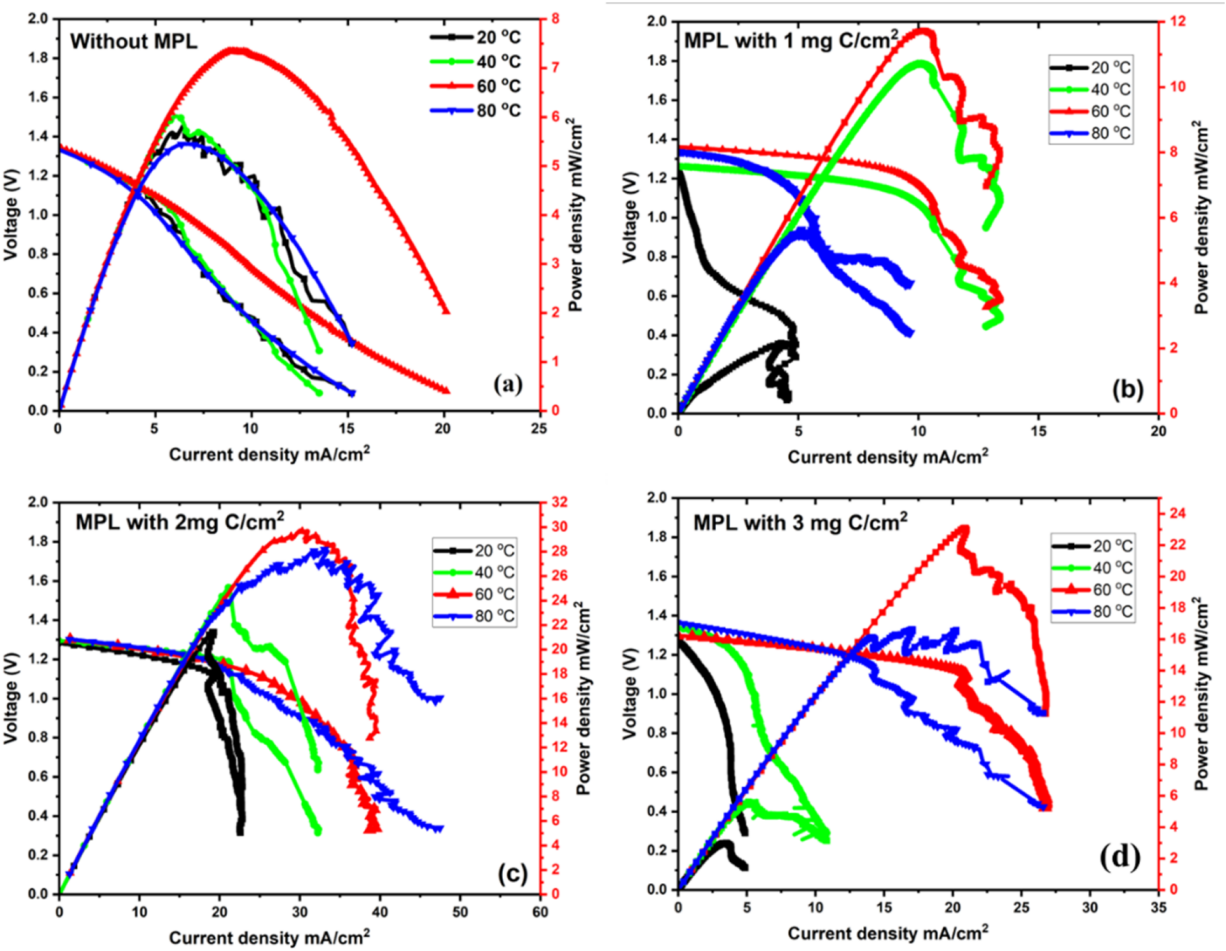
Polarization and power density curves for H_2_/Cl_2_ fuel cell mode at different operating temperatures (20 °C, 40 °C, 60 °C, and 80 °C) using anodic electrodes with MPL with carbon loading of (a) no MPL, (b) 1 mg of C/cm^2^, (c) 2 mg of C/cm^2^, and (d) 3 mg of C/cm^2^.

Moreover, the activation loss region was significantly less pronounced than that in typical H_2_/O_2_ fuel cells. This improvement is attributed to the relatively fast kinetics of the chlorine reduction reaction on the ruthenium oxide-based catalyst. The Cl_2_/Cl^–^ redox couple exhibits an exchange current density around 1 × 10^–2^ mA/cm^2^, compared to 1 × 10^–6^ mA/cm^2^ for the oxygen reduction reaction on platinum.^45^ The higher exchange current density leads to lower overpotentials, faster electrode kinetics, and improved energy conversion efficiency, particularly at low current densities.^46,47^

Figure 11 illustrates the impact of carbon loading in the microporous layer (MPL), now positioned on the cathode side, on the performance of the H_2_/Cl_2_ fuel cell. The data clearly show that MPL plays a significant role in enhancing power output. At 60 °C, the cell without an MPL achieved a peak power density of approximately 7 mW/cm^2^, consistent with previous findings by our group.^39^ However, introducing a 2 mgC/cm^2^ loading in the MPL led to a substantial increase in power output, reaching ∼30 mW/cm^2^, an order of magnitude improvement, and the highest performance recorded to date. Interestingly, further increasing the carbon loading to 3 mg C/cm^2^ resulted in a decline in power output to around 23 mW/cm^2^, indicating a performance threshold beyond which additional carbon may hinder efficiency. This significant improvement when adding the carbon up to 2 mg C/cm^2^ can be attributed to the MPL’s ability to enhance gas diffusion and water management, which facilitates more efficient reactant access and product removal at the electrode interface. The MPL also contributes to the formation of a more stable three-phase boundary, which is essential for effective electrochemical reactions.^14^ Moreover, hydrophobic MPLs reduce mass-transport resistance and alleviate flooding issues in the fuel cell.^34,48^ Additionally, the MPL supports better electrical connectivity by ensuring consistent contact between the catalyst and Ti felt layer, reducing charge-transfer resistance by enhancing water/gas management and electrical performance^49^ as it was demonstrated in Figure 2.

The reduction of the output power at the highest content of carbon in the MPL (3 mgC/cm^2^) suggests that beyond a certain thickness, the MPL may begin to hinder gas transport or introduce additional resistance by blocking the gas-phase channel or reducing porosity.^50^ Additionally, excess MPL thickness can trap liquid water and increase saturation near the catalyst interface.^51^ The thicker MPLs reduced breakthrough pressure for water droplets, increasing flooding in fuel cells, and negatively impacting gas accessibility.^52^

Figure 11 and Supporting Figure S5 clearly demonstrate the influence of operating temperature on cell efficiency, voltage characteristics, and peak power density. At lower temperatures (20 °C–40 °C), all electrode configurations exhibited diminished current densities and reduced power output. This performance degradation is primarily attributed to sluggish electrochemical kinetics and decreased ionic conductivity under these conditions, which collectively contribute to increased ohmic and activation overpotentials. Moreover, gas bubble detachment is less efficient at low temperatures, leading to blockage at the electrode–electrolyte interface and increased mass transport resistance. As the operating temperature increased to 60 °C, a significant improvement in cell performance was observed for all tests, particularly when an amount of 2 mg C/cm^2^ and 3 mg C/cm^2^ of carbon was deposited onto the electrode. The voltage profiles exhibited lower initial drops, and the maximum power density increased substantially. These enhancements are primarily due to the improved kinetics of both the hydrogen oxidation and chlorine reduction reactions at high temperatures as well as enhanced electrolyte conductivity by increasing the ion mobility and reactant/product transport at the electrode surfaces.

At 80 °C, the fuel cell exhibited reduced and unstable power output compared to the performance at 60 °C. This behavior may be attributed to thermal degradation or, most likely, to the evaporation of Cl_2_ species at elevated temperatures, which could diminish the availability of active oxidants and disrupt local pH conditions. A similar trend was observed in our previous study^39,42^

The temperature-dependent behavior observed in Figure 11 arises from a dynamic interplay between kinetic and ohmic enhancements and mass transport limitations. This balance is modulated by both the microporous layer (MPL) thickness and the thermodynamic properties of chlorine species, which together influence reactant accessibility and electrochemical performance under varying thermal conditions. As the temperature increases, charge-transfer and ohmic losses drop (see SI, Figure 6), which favors higher power. Elevated temperatures enhance reaction kinetics and improve ionic conductivity across the membrane. However, thermal stress, particularly in the presence of chlorine species, can compromise membrane integrity. Heat can induce swelling and chemical degradation weaken the membrane structure, while increased volatilization of chlorine and hypochlorous acid (HOCl) depletes local water and reactant concentrations. This leads to membrane dehydration, reduced ion mobility, and diminished fuel utilization efficiency.^53^

For thin MPLs (0–1 mg of C·cm^–2^), as shown in Figure 11a,b, the HClO concentration decreases at 80 °C due to reduced solubility and increased evaporation. Consequently, optimal performance is observed at 60 °C, followed by 40 °C. On the other hand, at the 3 mg C cm^–2^ MPL, the thicker and more hydrophobic nature leads to an increase in the effective transport thickness. At low temperatures (20–40 °C), reduced electrolyte diffusivity and increased viscosity hinder the transport of dissolved Cl_2_ across the electrode layers, resulting in diminished performance. Elevating the temperature to 60–80 °C enhances diffusivity, reduces viscosity, and improves oxidant delivery and bubble removal, thereby shifting the performance optimum to higher temperatures, 60 °C, and subsequently 80 °C for 3 mg C·cm^–2^ MPLs. At high oxidant concentrations, thick MPLs require elevated temperatures to overcome transport resistance, as lower temperatures fail to sustain adequate reactant delivery through the extended diffusion path.^54^

Figure 11 shows fluctuations in the polarization curves, with the MPL-free electrode displaying smoother behavior. Fluctuation intensity increases with MPL loading and is primarily attributed to oxidant-side dynamics rather than catalyst stability. Performance variability could be primarily linked to oxidant-side dynamics rather than to catalyst degradation. Specifically, localized oxidant concentrations near the cathode diminished due to the accelerated decomposition and speciation shifts of hypochlorous acid (HOCl) at elevated temperatures [65 °C]. Additionally, minor HOCl crossover through the membrane could occur, occasionally inducing transient voltage drops. The presence of a thicker, more hydrophobic microporous layer (MPL) further extended the oxidant transport pathway, thereby amplifying the magnitude of these fluctuations

In summary, the optimal performance of the H_2_/Cl_2_ fuel cell in this study was achieved at 60 °C with a carbon loading of 2 mg of C/cm^2^ in the MPL. This configuration provided a balance between enhanced electrochemical kinetics and thermal stability. These findings highlight the critical role of thermal management and electrode architecture in maximizing the efficiency of H_2_/Cl_2_ fuel cells.

Despite the improvements in catalyst loading and electrode design, the H_2_/HClO cell still delivers lower power than typical H_2_/air PEMFCs, which usually achieve around 0.5–1 W cm^–2^.^55^ This gap mainly reflects limits of chlorine chemistry and the cell architecture: chlorine species are highly soluble and reactive, creating mass-transport constraints and parasitic side reactions, while their corrosivity can depress catalyst activity and durability.^56^ Although RuO_2_ is very active for the chlorine evolution reaction (CER), the lower power observed suggests that the chlorine reduction reaction (CRR) is less active under our conditions.^46^ In addition, chlorine/hypochlorite redox couples can generate parasitic currents and Cl_2_/Cl^–^ crossover to the H_2_ side can induce mixed potentials and extra losses. To increase power density, future work should improve catholyte delivery and removal (higher flow, interdigitated channels, modify the cell design), develop chlorine-resistant and more active CRR catalysts, optimize ionomer content and distribution in thinner, more uniform catalyst layers, use reinforced high-conductivity membranes with barrier layers to limit crossover and contact resistance, and operate within a controlled window (∼50–60 °C, adequate hydration, and pH that stabilizes HClO). These steps should help narrow the performance gap.

### 3.10 CO_2_ Fixation Capacity in the Electrolysis Mode

In addition to hydrogen and chlorine production and the production of power, EDEN technology provides an advantage in enabling CO_2_ capture through electrochemically assisted alkaline absorption. During electrolysis, hydroxide ions (OH^–^) are generated at the cathode, which can react with CO_2_ to form bicarbonates (HCO_3_^–^) or carbonates (CO_3_^2–^), as shown in eqs 7 and 8. This transformation supports the integration of CO_2_ fixation into the electrochemical system:

7 $${\mathrm{CO}}_{2}+{\mathrm{OH}}^{-}\to {{\mathrm{HCO}}_{3}}^{-}$$

8 $${\mathrm{CO}}_{2}+2{\mathrm{OH}}^{-}\to C{O_{3}}^{2-}+H_{2}O$$

According to these equations, an increase in pH reflects enhanced hydroxide availability and, consequently, a greater potential for CO_2_ capture through bicarbonate or carbonate formation.

Our system, previously validated for this application, demonstrates that under average operating conditions, approximately 1.218 mol of NaOH are required to capture each mole of CO_2_^3^,^41^ reflecting the mixed formation of both HCO_3_^–^ and CO_3_^2–^ species. The more pronounced pH increase observed at higher temperatures and with a carbon loading of 2 mg C/cm^2^ (see Supporting Figure S6) suggests enhanced NaOH production efficiency. This improvement directly correlates with the system’s increased capacity for CO_2_ absorption, as illustrated in Figure 12.

**Figure 12:**
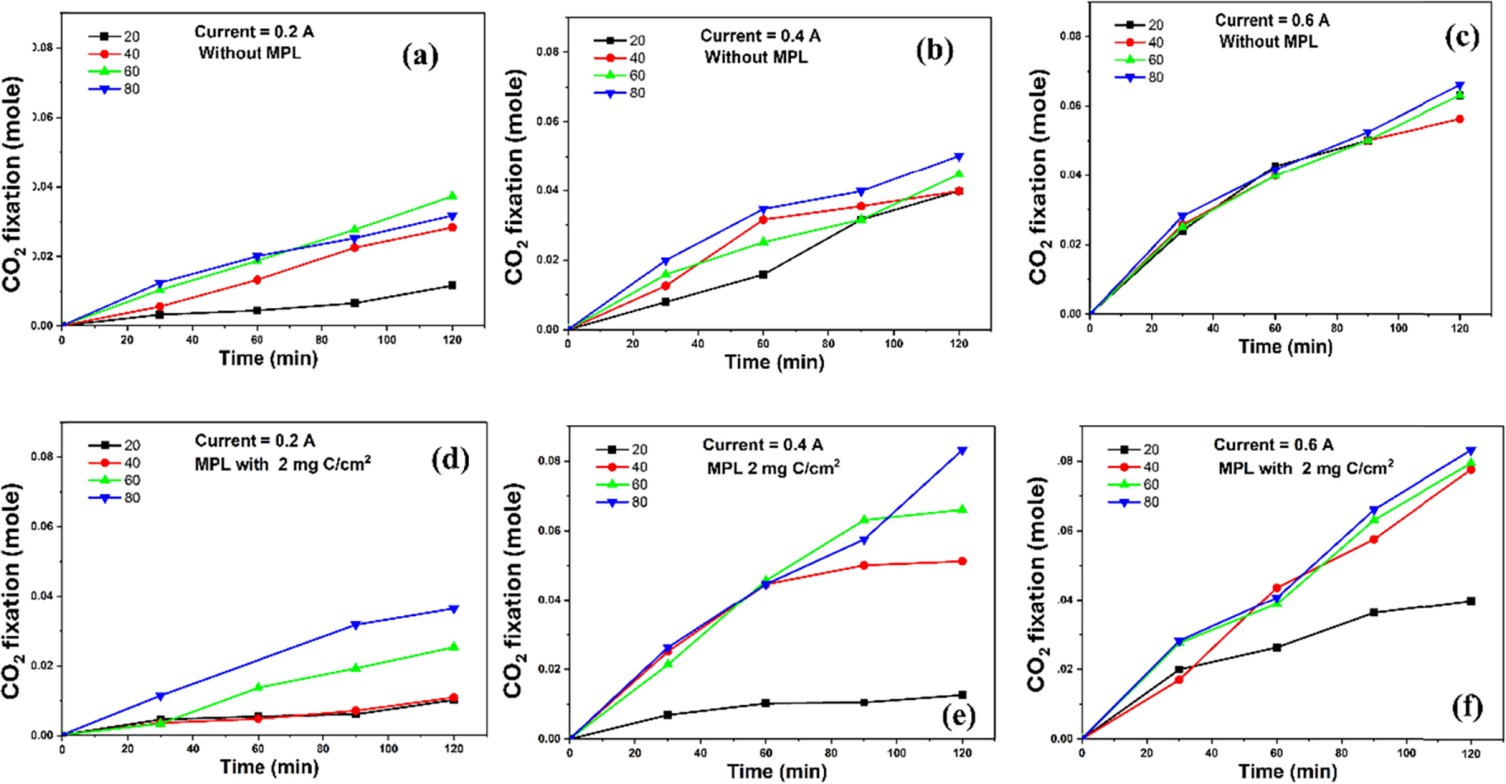
Estimated CO_2_ fixation capacity (mole) as a function of electrolysis duration at different temperatures (20 °C, 40 °C, 60 °C, 80 °C), applied currents, and different carbon laoding in MPL: (a) no MPL at 0.2A, (b) no MPL at 0.4A, (c) no MPL at 0.6A, (d) 2 mg C/cm^2^ at 0.2A, (e) 2 mg C/cm^2^ at 0.4A, and (f) 2 mg C/cm^2^ at 0.6A.

These findings indicate that electrochemical parameters influence not only gas evolution efficiency but also the system’s environmental sustainability. Specifically, operating at moderate temperatures and lower current densities promotes both electrochemical performance and environmental functionality by maximizing the hydroxide generation and subsequent CO_2_ fixation.

## 4 Conclusions

Titanium felt electrodes, modified with varying carbon content in the microporous layer and coated with a RuO_2_–Pt catalyst via a Pechini-type polymeric precursor method, were evaluated under both electrolysis and H_2_/Cl_2_ fuel cell operating modes. A carbon loading of 2 mg C/cm^2^ in the MPL was identified as the optimal configuration, based on electronic conductivity and hydrophobicity measurements.

In electrolysis mode, the system achieved a high hydrogen production efficiency of 15 mgH_2_/Wh at 60 °C, surpassing several industrial benchmarks. In fuel cell mode, the same optimized MPL configuration delivered a peak power density of approximately 30 mW/cm^2^, which is the highest reported for this system to date. However, excessive carbon loading in the MPL led to performance degradation due to mass transport limitations.

Additionally, the system demonstrated enhanced Faradaic efficiency (>98%) and the capability to facilitate CO_2_ capture via cathodic alkaline absorption, underscoring the multifunctionality and practical potential of the designed electrochemical architecture.

Overall, these findings highlight the critical role of electrode architecture and thermal management in the advancement of integrated energy systems. The results position chlor-alkali-based reversible electrochemical cells as a promising platform for efficient, scalable, and multifunctional energy storage and conversion technologies. Future work will focus on operando probing of the potential distribution within the microporous layer to confirm the maintenance of benign electrochemical conditions for the carbon phase. This, alongside extended durability testing exceeding 1000 h under dynamic load, will serve as the ultimate validation for the practical application of this DSA-based electrode architecture in unitized reversible energy storage systems. These efforts aim to further advance the overall energy efficiency of this emerging electrochemical system. Overall, these results position chlor-alkali-based reversible cells as a promising and multifunctional platform for efficient, scalable energy storage and CO_2_ capture.

## Supplementary Material



## Notes

Average cell voltage value as a function of different carbon loadings under constant current operation at different temperature; hydrogen production profiles over time at different applied current values and operating temperatures; comparison between the theoretical and experimental hydrogen production profiles over time at different temperatures; hydrogen energy efficiency (mgH_2_/Wh); temperature dependence of peak power density in fuel cell mode; the change of catholyte pH under various operating temperatures and applied current densities for electrolysis; and ohmic resistance and charge-transfer resistance values at different temperatures (PDF)
