# Supplementary material for: To Carbon or Not to Carbon: Rethinking Electrode Design in Unitized Reversible Fuel Cells
Source: ACS Appl Mater Interfaces. 2026 Mar 3;18(10):14862–78. doi: 10.1021/acsami.5c15144 (PMC13006945; doi:10.1021/acsami.5c15144)
Supplement: Supplementary file 1 [file am5c15144_si_001.pdf]

Supporting Information

To Carbon or Not to Carbon: Rethinking Electrode Design in Unitized Reversible Fuel Cells

Mahmoud M. Gomaa[a,b], Prince S.A. Nopuo[b], Manuel Andrés Rodrigo[b] and Justo Lobato*[b]

[a] Physics Department, Faculty of Science, Minia University, P.O. Box 61519 Minia, Egypt [b] Chemical Engineering Department. Enrique Costa Novella building. Av. Camilo José Cela 12. University of Castilla-La Mancha, 13004, - Ciudad Real, Spain

*Corresponding author: justo.lobato@uclm.es

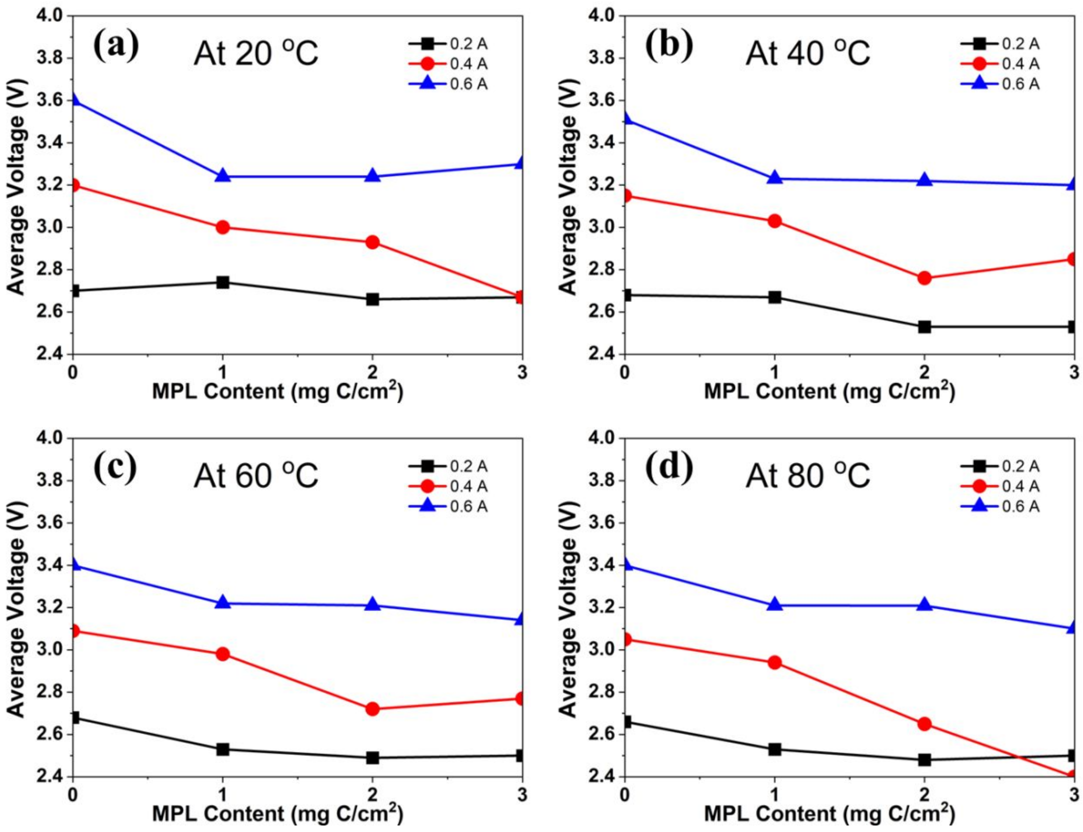

Figure S1. Average cell voltage value as a function of different carbon loadings (0, 1, 2, and 3 mg C/cm²) under constant current operation (0.2 A, 0.4 A, and 0.6 A) at different temperature. The values were obtained from the time-resolved electrolysis data shown in Figure 7.

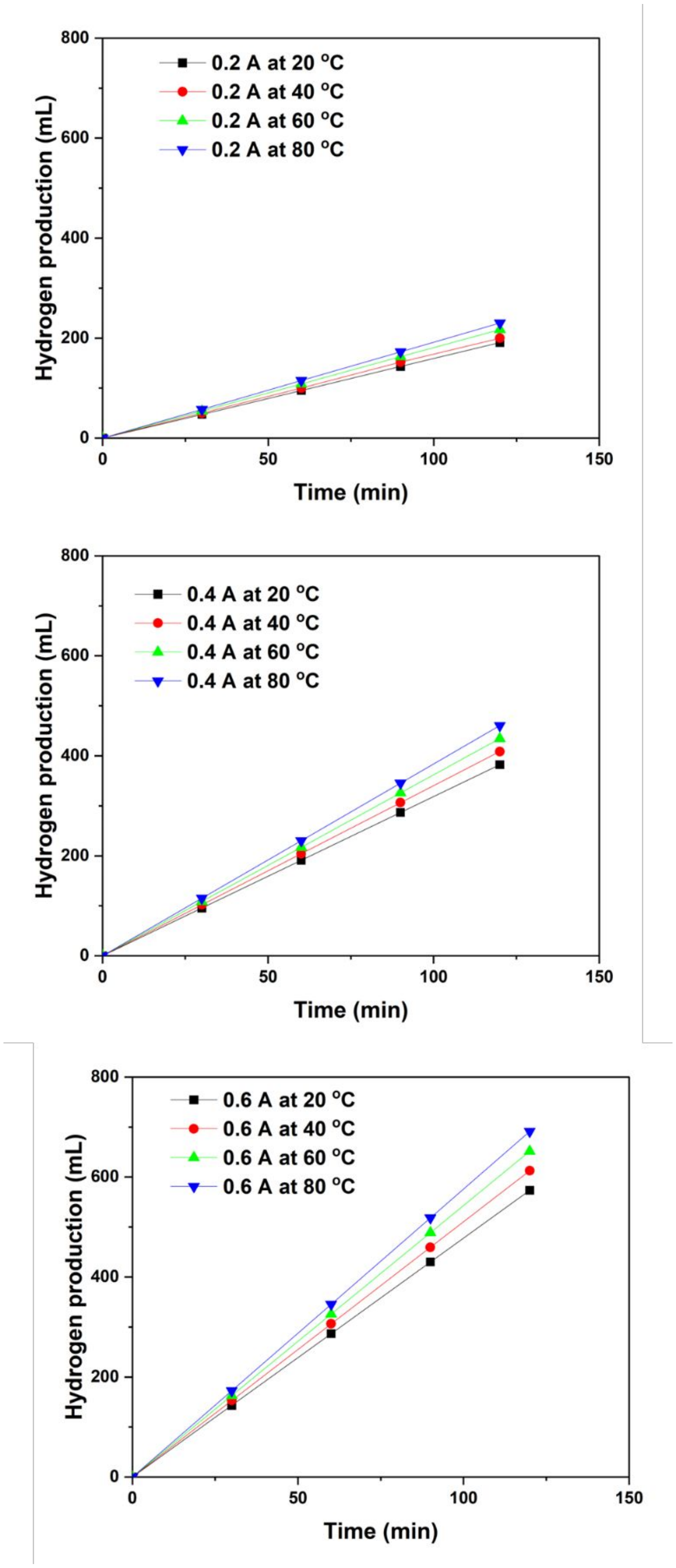

Figure S2: Hydrogen production profiles over time at different applied current values (0.2 A, 0.4 A, 0.6 A) and operating temperatures (20°C, 40°C, 60°C, 80°C) using 2 mg C/cm^2 of MPL.

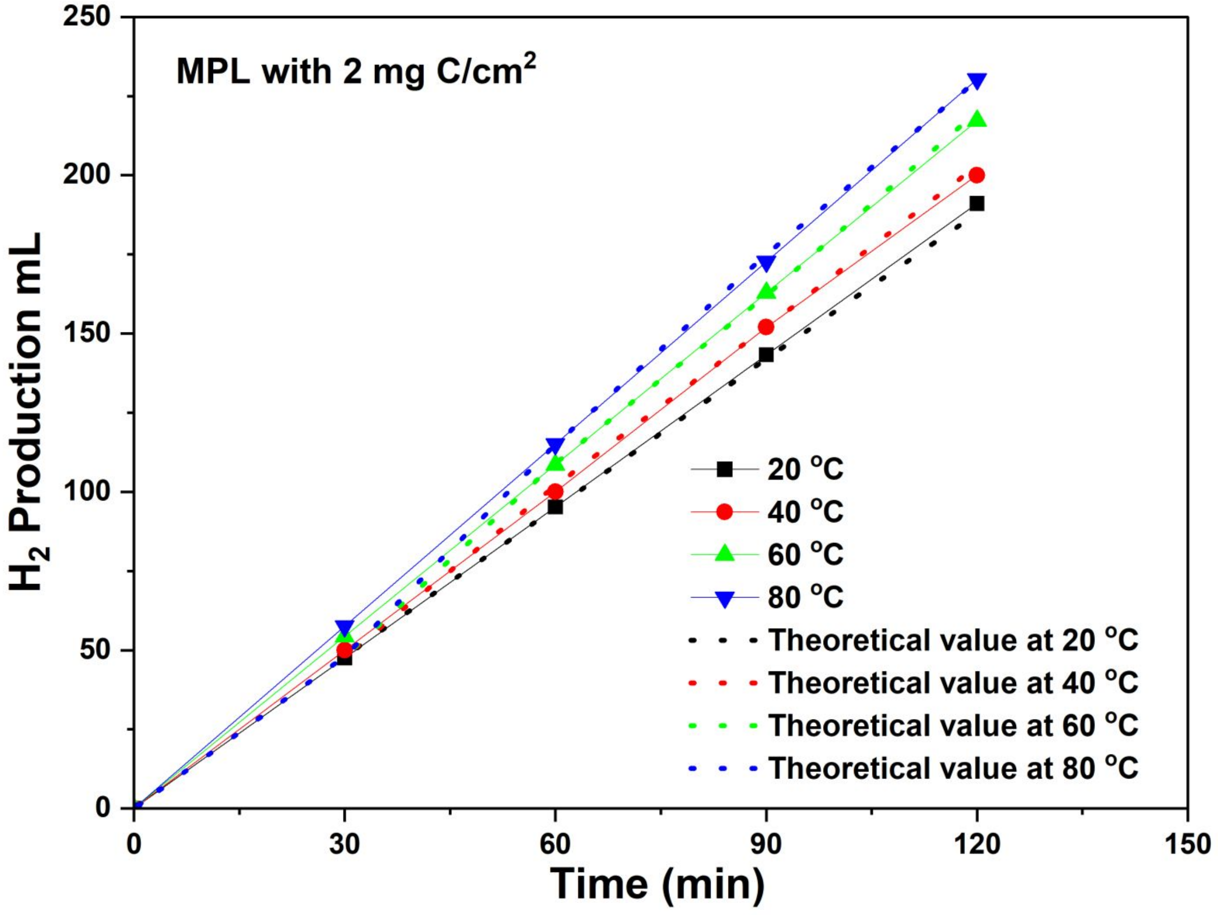

Figure S3. Comparison between the theoretical and experimental hydrogen production profiles over time at different temperatures, using a constant current of 0.2 A and an MPL loading of 2 mgC/cm².

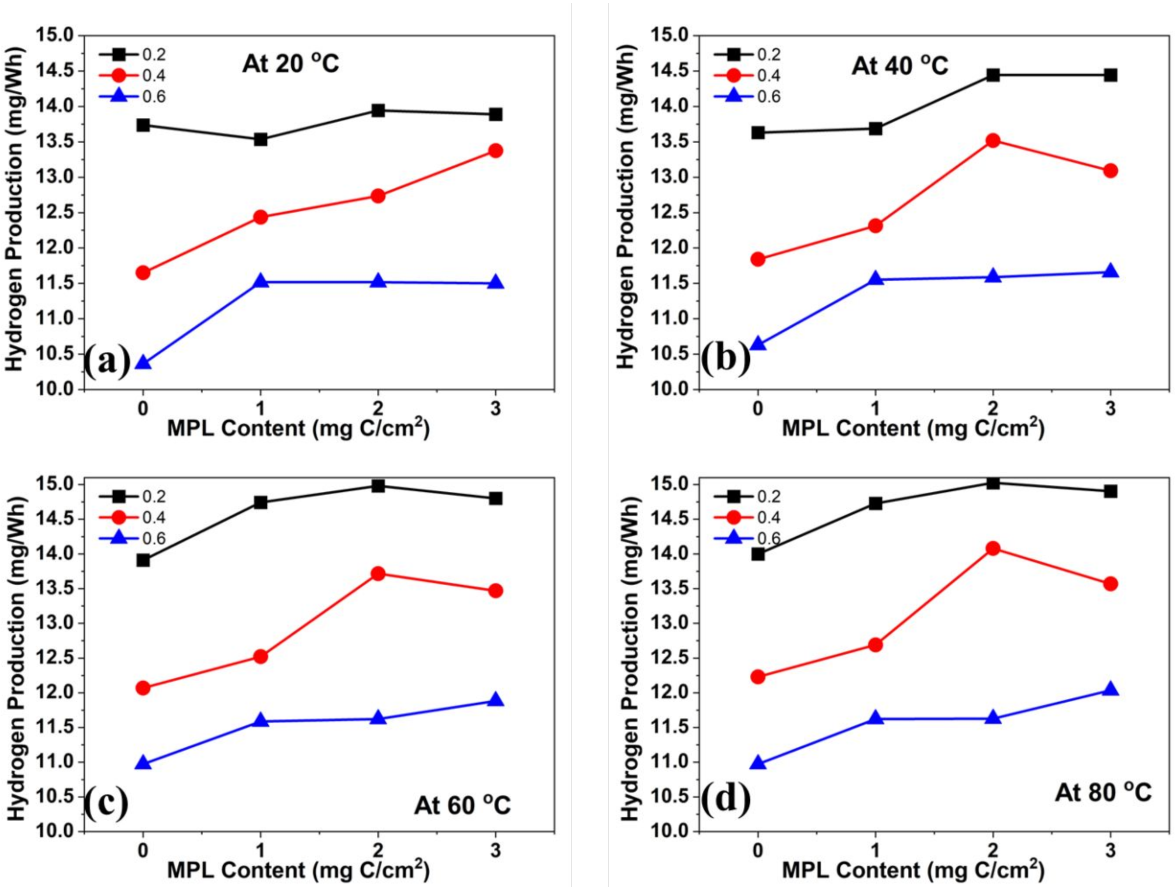

Figure S4. Hydrogen energy efficiency (mgH_2/Wh) at different carbon contents in the MPL, different temperatures and at different current densities.

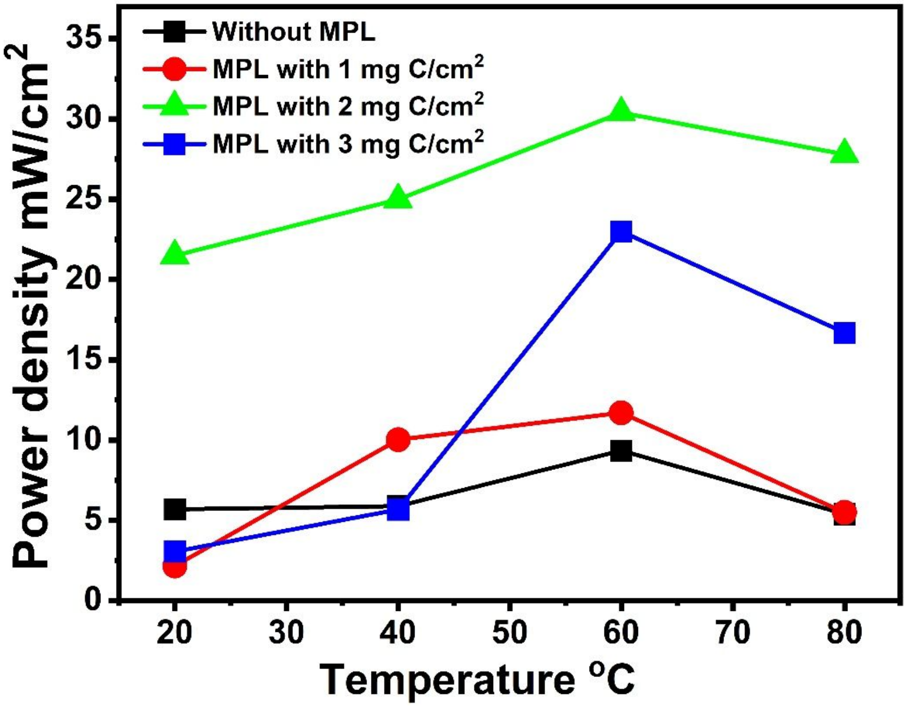

Figure S5. Temperature dependence of peak power density in fuel cell mode using electrodes without MPL and with MPL carbon loadings of 1, 2, and 3 mg C cm^{-2}.

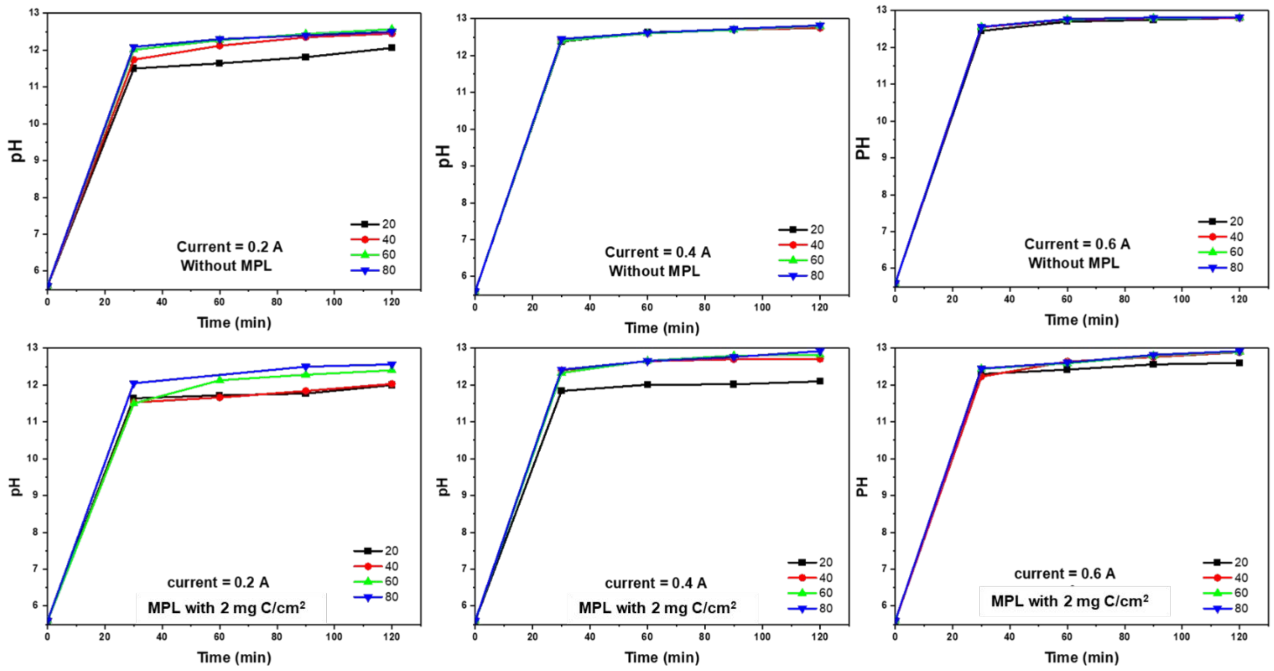

Figure S6: The change of catholyte pH under various operating temperatures (20°C, 40°C, 60°C, 80°C) and applied current densities (0.2 A, 0.4 A, 0.6 A) for electrolysis using (top row) without MPL and (bottom row) Ti felt coated with 2 mgC/cm² in the MPL.

Table S1. Ohmic resistance and charge-transfer resistance at different temperatures for Ti felt electrodes with MPL carbon loadings of 0, 1, 2, and 3 mg C cm^{-2}.

	Ohmic Resistance R (Ω)				Charge Transfer Resistance Rct (Ω)			
**T (°C)**	**No MPL**	**MPL with 1 mg C·cm^{-2})**	**MPL with 2 mg C·cm^{-2})**	**MPL with 3 mg C·cm^{-2})**	**No MPL**	**MPL with 1 mg C·cm^{-2})**	**MPL with 2 mg C·cm^{-2})**	**MPL with 3 mg C·cm^{-2})**
**20**	1.370	1.199	0.954	0.818	0.508	0.394	0.298	0.185
**40**	1.324	1.175	0.830	0.649	0.390	0.366	0.150	0.122
**60**	1.270	1.152	0.824	0.636	0.407	0.385	0.100	0.116
**80**	1.236	1.123	0.886	0.687	0.326	0.237	0.160	0.257
